# PROTAC’ing oncoproteins: targeted protein degradation for cancer therapy

**DOI:** 10.1186/s12943-022-01707-5

**Published:** 2023-03-30

**Authors:** Jeremy M. Kelm, Deepti S. Pandey, Evan Malin, Hussein Kansou, Sahil Arora, Raj Kumar, Navnath S. Gavande

**Affiliations:** 1 Department of Pharmaceutical Sciences, Eugene Applebaum College of Pharmacy and Health Sciences (EACPHS), Wayne State University, Detroit, MI 48201 USA; 2 Laboratory for Drug Design and Synthesis, Department of Pharmaceutical Sciences and Natural Products, Central University of Punjab, Bathinda, 151401 India; 3 Molecular Therapeutics Program, Barbara Ann Karmanos Cancer Institute, Wayne State University School of Medicine, Detroit, MI 48201 USA

## Abstract

Molecularly targeted cancer therapies substantially improve patient outcomes, although the durability of their effectiveness can be limited. Resistance to these therapies is often related to adaptive changes in the target oncoprotein which reduce binding affinity. The arsenal of targeted cancer therapies, moreover, lacks coverage of several notorious oncoproteins with challenging features for inhibitor development. Degraders are a relatively new therapeutic modality which deplete the target protein by hijacking the cellular protein destruction machinery. Degraders offer several advantages for cancer therapy including resiliency to acquired mutations in the target protein, enhanced selectivity, lower dosing requirements, and the potential to abrogate oncogenic transcription factors and scaffolding proteins. Herein, we review the development of proteolysis targeting chimeras (PROTACs) for selected cancer therapy targets and their reported biological activities. The medicinal chemistry of PROTAC design has been a challenging area of active research, but the recent advances in the field will usher in an era of rational degrader design.

## Introduction

### Cancer and current treatment paradigms

Cancer refers to a group of diseases that are initiated by genetic and epigenetic aberrations that present clinically as uncontrolled cell growth and metastasis. In the United States, cancer is the second-leading cause of death with lung, prostate, breast, and colorectal cancers being most common [1]. The paradigms of existing cancer pharmacotherapy can be broadly summarized to include (i) agents that selectively kill rapidly dividing cells by inducing DNA damage, replication stress, or by disrupting the cytoskeleton; (ii) agents which block sex hormone receptors or deplete their activating ligands; and (iii) molecularly targeted therapies which disrupt tumor growth, blood supply, or interfere with evasion of apoptosis and the immune system [2–11]. In the past two decades, the majority of newly approved cancer therapies fall into the third category of molecularly targeted therapies. These advances have contributed to the aversion of 4.5 million cancer deaths between 1991 to 2019 [1].

Unfortunately, the available molecularly targeted therapies still face a number of challenges which limit both the durability of their efficacy and their broad applicability. At the core of the issue is that with few exceptions, existing molecularly targeted therapies operate by an occupancy-based mechanism of action (MOA). Specifically, occupancy-based therapeutics only exert their activity on the target while in the bound state. This translates into a requirement for dosing that is sufficiently high and frequent to continually saturate the vast majority of the copies of the target. While this approach is often initially efficacious, adaptive resistance mechanisms including target overexpression, gene amplification, drug efflux, and point mutations involving the drug’s binding site increase the dose required to saturate the target sufficiently [12]. Effectively, these adaptive resistance mechanisms narrow or eliminate the therapeutic index of the drug because higher drug concentrations often result in off-target toxicities. Occupancy-based inhibitors implicitly rely upon high binding affinity and are thus vulnerable to point mutations involving the drug binding site [11, 12]. In many cases, the application of cancer treatment is what drives loss of the ligand binding site or point mutations which prevent binding [13–19]. These are expected outcomes for oncoproteins harbored by cancer cells facing natural selection and genomic instability. Target-independent resistance mechanisms including compensation by other pathways is an additional challenge for the field of molecular cancer therapeutics [12].

The requirement for high affinity binding limits the applicability of occupancy-based inhibitors to many oncoproteins that reside in what is often referred to as the dark or undruggable proteome [20–22]. The undruggable oncoprotein designation is typically reserved for targets displaying one or several challenging features: (i) nonenzymatic functions, (ii) lack of deep hydrophobic pockets, and (iii) high affinity for an abundant substrate. While there have been considerable advances in drugging protein-protein interactions (PPIs), modulators of these interactions remain challenging to develop [23]. For many relevant PPIs, the contact area exceeds 1500 Å^2^, involves pocketless surfaces, and the surface geometry is dynamically influenced by other binding partners [20, 23]. The oncogenic roles of PPIs for an enzymatic target are sometimes only apparent after occupancy-based approaches yield disappointing results [24]. In other cases, residual signaling despite inhibitor binding or depletion of the activating ligand is sufficient to drive continued tumor growth [25, 26]. Collectively, these observations suggest that leaving the target intact and the requirement for high binding affinity are concerning liabilities of occupancy-based inhibitors for cancer therapy targets.

Compounds which induce proteasomal degradation are less sensitive to losses of affinity and implicitly prevent target PPIs and breakthrough signaling [27]. Accordingly, degraders are well-suited to address some of the challenges associated with abrogating oncoproteins. While only several FDA approved therapies employ a degrader MOA, targeted protein degradation (TPD) as a therapeutic modality has garnered enormous interest in recent years [28]. Herein, we review the challenging aspects of the development of heterobifunctional proteolysis targeting chimeras (PROTACs) for selected cancer therapy targets and their reported biological activities. We additionally provide a review of the PROTAC platform and target pharmacology in the context of degrader rationale. Other therapeutic degrader platforms including molecular glues, LYTACs, and homobivalent- and trivalent-PROTACs have been reviewed separately [29–31].

### The ubiquitin proteasome system

Protein degradation is a natural cellular function that enables the clearance of denatured, mutated, and otherwise unneeded proteins [32]. In humans, the ubiquitin-proteasome system (UPS) achieves selective protein degradation in a highly regulated manner. Beyond removal of defective and unnecessary proteins, the UPS may be employed to orchestrate various cellular functions such as by degrading proteins that restrict cell cycle progression through anaphase [33]. Dysregulation of the UPS is thought to be implicated in ageing and neurodegenerative diseases [34, 35]. E3 ligase complexes are the adaptor proteins of the UPS that confer substrate-specific ubiquitylation. To this end, E3 ligases serve as adaptors for E2 ligases which are responsible for catalyzing the transfer of ubiquitin tags to the recruited substrate. E3 ligases may also complex with additional proteins that modulate substrate specificity, allowing for the recognition of various proteins by a single E3 ligase in a context-dependent manner [36]. Ubiquitin tags are typically ligated to the ε-amino group of surface lysine residues on the targeted protein (monoubiquitylation) or lysine residues of pre-existing ubiquitin tags (polyubiquitylation) [37]. Ubiquitin is a small seventy-six amino acid (AA) protein containing a c-terminal glycine that is used for ligation and seven lysine residues (sites K6, K11, K27, K29, K33, K48, and K63) for ubiquitin chain extension [37, 38]. K48-linked ubiquitin chains encode for 26S proteasomal degradation whereas other ubiquitylation patterns confer alternative fates for the tagged protein [37]. A cysteine residue in E2 ligase receives activated ubiquitin from E1 ligases via thioesterification. E1 ligase activates ubiquitin in a reaction requiring ATP and Mg^2+^ [39]. K48 linked polyubiquitylated proteins are recognized by the 19S regulatory subunit of the 26S proteasome, which then allows access to the 20S core catalytic subunit [40]. After gate opening, the targeted protein is unfolded, deubiquitylated, and proteolytically degraded. More in-depth biochemical explanations of the members of the UPS and their interactions have been provided elsewhere [39, 41].

### The PROTAC platform and general considerations for PROTAC development

**Fig. 1:**
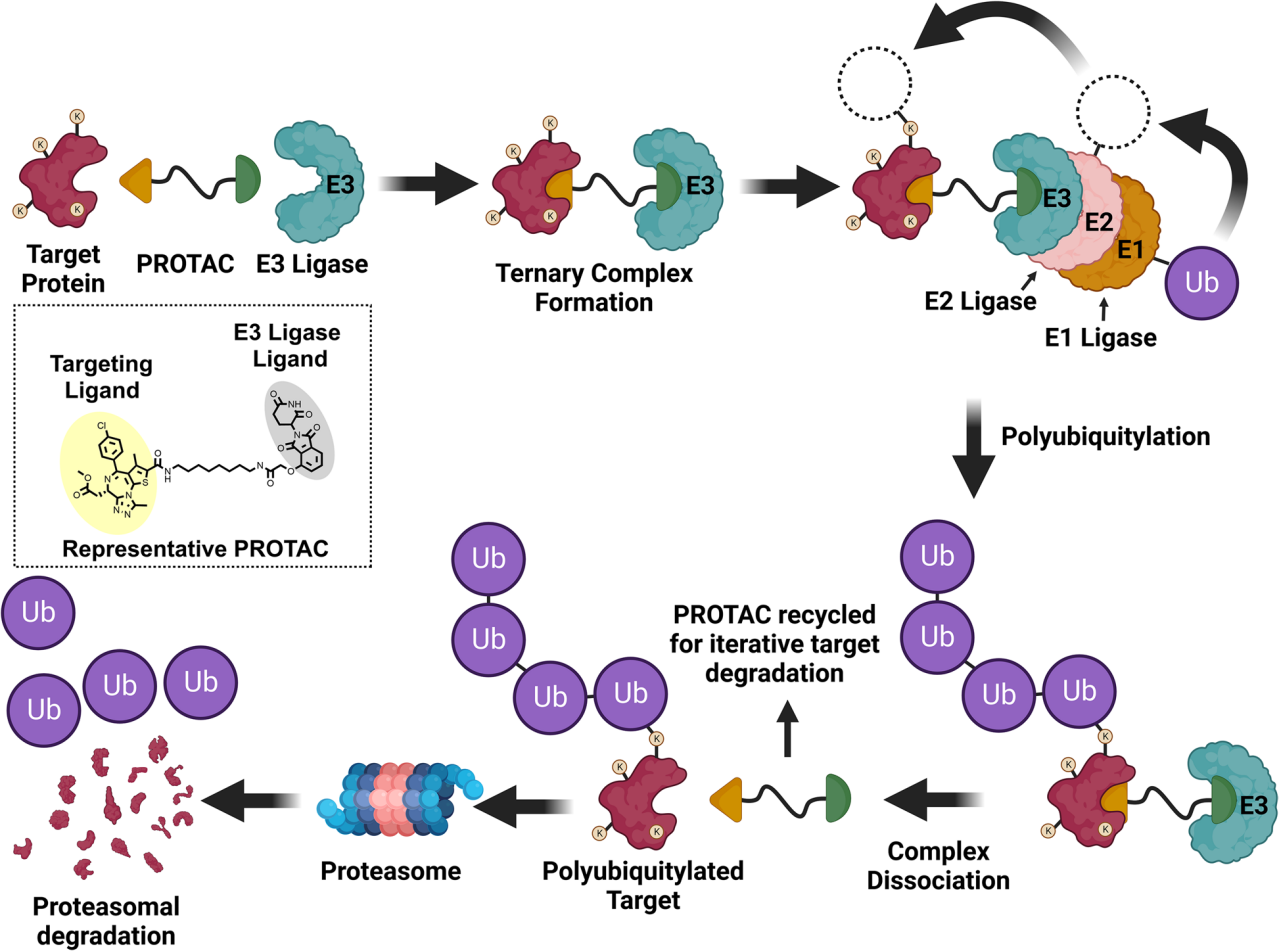
PROTACs induce catalytic proteasomal degradation of their targets. PROTACs are heterobifunctional compounds comprised of a ligand for a target protein and a ligand for an E3 ligase joined by a linker. Simultaneous binding of a target protein and an E3 ligase promotes the formation of ternary complexes: Target-PROTAC-E3 Ligase. E3 ligases serve as adaptor proteins for E2 ligases by conferring selective target recruitment. E2 ligases receive activated ubiquitin tags from E1 ligase and conjugate ubiquitin to surface lysine residues of the target protein. Ubiquitin tags may also be ligated to pre-existing ubiquitin tags to form polyubiquitin chains. Ternary complexes may dissociate after target ubiquitylation to enable iterative target degradation by a single PROTAC molecule. Polyubiquitylated targets are recognized by the 26S proteosome where they are degraded. Image created in Biorender and Chemdraw

PROTACs are heterobifunctional molecules that hijack the UPS by positioning a target protein of interest (POI) in proximity to an E3 ligase, resulting in ligation of a polyubiquitin chain to the POI (Fig. 1). The design strategy typically applied in the design of PROTACs involves appending a ligand, often a pharmacological inhibitor, of the POI to a ligand for an E3 ligase via a chemical linker [42]. The vital role of the linker is to orchestrate the appropriate geometry of the ternary complex comprised of the PROTAC, the POI, and the recruited E3 ligase. Upon formation of the ternary complex, ubiquitin tags are transferred from a recruited E2 ligase to the POI as described above [37]. The polyubiquitylated POI is then recognized by the 19S regulatory unit of the 26S proteasome where it is subsequently degraded. PROTACs are said to employ an event-driven MOA because proteasomal degradation of a polyubiquitylated POI can proceed following ternary complex dissociation. This is in contrast to occupancy-based inhibitors which only suppress POI activity while in the bound state. PROTACs are frequently referred to as being catalytic degraders because dissociation of the PROTAC from a polyubiquitylated POI allows for iterative target degradation (illustrated in Fig. 1). For this reason, PROTACs have the capability to degrade their respective targets at substoichiometric concentrations [43]. Summarily, PROTACs hijack the cellular protein destruction machinery to selectively remove targeted proteins and remain active at concentrations low enough to reduce off-target effects. In the remainder of this review, the terms “target” or “target protein” will be used to refer to the respective POI.

PROTACs provide an opportunity to significantly broaden the druggable proteome by degrading targets that are not suitable for occupancy-based inhibitors such as transcription factors, scaffolding proteins, and cytoskeletal proteins [44–46]. The PROTAC platform may also be particularly well-suited for degrading overexpressed oncoproteins because of their substoichiometric, catalytic MOA [43]. Moreover, targets bearing binding pockets that are conserved across related isoforms are challenging to approach with occupancy-based inhibitors because of nonselective binding [46, 47]. Such targets are highly suitable for a PROTAC-based medicinal chemistry campaign because of the additional layers of selectivity afforded by the platform. Here, selective target degradation can be achieved by exploiting differential (i) geometries of the ternary complexes and protein-protein contact interfaces and/or (ii) spatial patterning of surface lysine residues [48–50]. Residual off-target degradation can be mitigated by substoichiometric dosing unlike occupancy-based inhibitors which require target saturation [43, 51]. In 2021, Schneider et al. described a systematic approach to evaluating the tractability of targets by the PROTAC platform and provided a publicly-available data set of 1067 candidate targets for which no PROTACs had been reported [52].

High-jacking E3 ligases for TPD poses a theoretical concern for stabilizing the natural substrates of the recruited E3 ligase [43]. Accordingly, the therapeutic index of a PROTAC is sometimes reported which refers to the concentration required to stabilize the natural E3 ligase substrate (i.e., HIF-1α for VHL) divided by the concentration conferring half-maximal target degradation (DC_50_). As inducers of ternary complexes in three-body binding equilibria, PROTACs are implicitly liable to a hook effect [53]. In essence, supraoptimal PROTAC concentrations saturate both the target and corresponding E3 ligase, resulting in non-productive bivalent complexes (PROTAC-E3 Ligase and PROTAC-Target) rather than the desired ternary complex. Consequently, most PROTACs display an optimal concentration for target degradation that when exceeded affords reduced degradation [54].

An important consideration in the design of PROTACs is selection of an E3 ligase complex to be targeted for recruitment by the PROTAC. While the E3 ligase family comprises over 600 distinct proteins, only a handful of them have been successfully targeted for recruitment by PROTACs [55–57]. Discovery of suitable ligands for additional E3 ligases is an area of active research [56, 57]. The vast majority of PROTACs reported recently include a ligand that recruits either the Von Hippel Lindau protein (VHL) or cereblon (CRBN). While the preferred E3 ligase may depend on the identity of the target protein, some experts have described VHL-dependent PROTACs as generally requiring less extensive linker geometry optimization than their CRBN-dependent counterparts [58]. Contrarily, CRBN ligands confer lower molecular weight (MW) and better drug-like properties [58]. An additional consideration relevant to E3 ligase ligand selection is the relative expression and functionality of the various E3 ligases in the cells harboring the pharmacological target. In 2022, Luo et al. examined the potency of MZ1 and dBET1 (two well-characterized BRD4 degraders that differ only by E3 ligase ligand) across fifty-six cancer cell lines representing ten cancer subtypes [59]. This report discovered that low expression or inactivating mutations of CRBN/VHL predict a poor response to the corresponding CRBN/VHL-recruiting PROTAC. As ligands are developed for additional E3 ligases, medicinal chemists may opt for selection of the E3 ligase whose structural characteristics predict strong cooperative binding with the target protein in the ternary complex [60, 61]. Promoting cooperative interactions between the target and the E3 ligase is one such strategy to increase PROTAC potency and minimize the hook effect associated with the platform [62]. Others have found that for some targets thermodynamic cooperativity is not strictly required, although alleviation of steric clashes between the two substrates in the ternary complex is decisive [50, 63, 64]. While examining the cocrystal structures of PROTAC ternary complexes, Nowak et al. observed that subtle changes in linker composition induce disparate sets of interprotein interactions between the E3 ligase and the target protein [50]. Therein, methods for modeling PROTAC ternary complexes and accompanying PPIs using Rosetta were described.

**Fig. 2:**
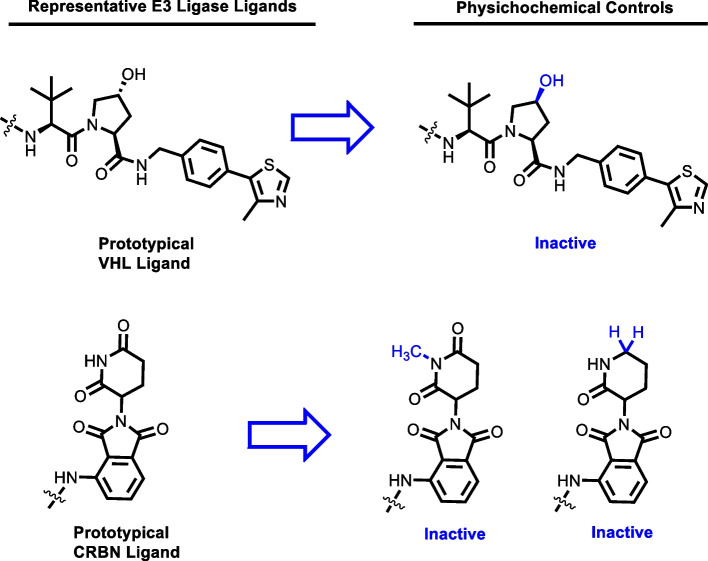
Prototypical CRBN and VHL ligands and their respective physicochemical controls. Epimerization of the chiral proline ring of VHL ligand prevents VHL recruitment. The *N*-methyl and descarbonyl congeners of the CRBN ligand cannot recruit CRBN

The VHL protein is a member of the VHL E3 ligase complex (VBC) comprised of cullin 2, elongins B and C, and Rbx-1 [65, 66]. In cellular physiology, the VHL protein acts as a tumor suppressor by targeting hypoxia-inducible factors (HIFs) for degradation. Ligands for VHL were derived by modification of a small peptide fragment from HIF-1α [67, 68]. Interestingly, VHL is expressed minimally in platelets, which may allow for tumor-selective degradation of targets classically associated with producing thrombocytopenia when inhibited [69]. Negative controls of VHL-dependent PROTACs can be acquired by epimerization of the chiral hydroxyl group in the proline ring (Fig. 2). The second commonly recruited protein in PROTAC design, CRBN, complexes with CUL4, RBX1, and DDB1 to yield the CRL4 E3 ligase complex [70]. CRBN appears to play a role in nervous system development during embryogenesis, as mutations of the CRBN gene result in mental retardation [71]. Ligands for CRBN that are exploited in the design of PROTACs are typically thalidomide, lenalidomide, pomalidomide, among other phthalimide derivatives. The discovery of these ligands for CRBN was serendipitous to the discovery of thalidomide’s capacity to ubiquitylate Ikaros transcription factors by forming a cryptic interface on CRBN [70, 72]. The cocrystal structure of *g. gallus* CRBN and thalidomide was reported in 2014 by Fischer et al. and has had important implications for the design of CRBN-recruiting PROTACs [70]. Negative controls of CRBN-dependent PROTACs can be generated by incorporating *N-*methyl or descarbonyl congeners of the glutarimide ring (Fig. 2). Other E3 ligases targeted for recruitment by PROTACs reviewed herein include Mdm2, SCF, cIAP1, and RNF114. In normal cellular physiology, MDM2 is the E3-ligase responsible for regulating cycle progression by targeting p53 for destruction and can be recruited with imidazoline-based ligands [73, 74]. Chimeric degraders that recruit cIAP1 as the selected E3 ligase are commonly referred to as specific and nongenetic IAP-dependent protein erasers (SNIPERs). A limitation of the SNIPER platform is that cIAP1 ligands have been reported to induce auto-ubiquitylation and proteasomal degradation of the recruited cIAP1 molecule [75]. RNF114 is an E3 ligase with roles in T-cell activation, psoriasis, and male fertility that can be artificially recruited with the natural product nimbolide [76–79]. Alternative E3 ligases suitable for recruitment by the PROTAC platform and the discovery of their corresponding ligands is reviewed in detail elsewhere [56, 57].

Elucidating linker structure-degradation relationships (SDRs) for a pair of target- and E3 ligase-recruiting ligands is typically done empirically and thus requires intensive chemical synthesis [80, 81]. The first matter to be sorted with regard to the linker is where on the selected ligands it is best ligated. A solvent-exposed region of each ligand is typically utilized as the point of linker attachment so as to not disturb binding affinity. The identification of the solvent-exposed groups requires an available cocrystal of the ligand and its binding partner or computational analysis. Having determined suitable positions for linker attachment, a generic strategy in the design of a PROTAC linker is to first determine an optimal linker length via use of polyethylene glycol (PEG) or linear alkyl chains, with or without incorporated amide groups. However, a recent systematic review of reported PROTACs suggests that exploratory syntheses focused on longer PEG and alkyl linkers may be the most efficient medicinal chemistry strategy [81]. This strategy enables the investigator to promptly gauge target-E3 ligase compatibility and linker composition preferences without the confounding impact of steric clashes [81]. After identifying a range of suitable linker lengths, the chemical composition of the linker as well as its flexibility is modulated to produce a finely-tuned balance of target degradation, cellular permeability, and aqueous solubility. The incorporation of azaheterocycles in the linker is an evolving trend in PROTAC design owing to their influence on rigidity, cellular permeability (potentially via lysosomal uptake), and aqueous solubility [82, 83]. Phenoxyethers are an alternative class of heterocycles that have been utilized in linker design [84]. Click chemistry has been utilized to expediently synthesize triazole-containing PROTACs from the corresponding azides and alkynes [85]. In 2022, a resin-scavenging direct to biology (D2B) strategy was described which enabled high-throughput synthesis and biological assessment of crude PROTAC mixtures without chromatography [86].

### Early PROTACs

The concept of targeted ubiquitylation and subsequent degradation dates back to a report in 1995 by Gosink & Vierstra wherein they directed in vitro degradation of various protein targets using *E. coli* engineered to express recombinant E2 ligases [87]. Recombinant E2 ligases were constructed such that inserted C-terminal sequences allowed for selective neosubstrate recruitment by the E2 ligase. The inserted C-terminal fragment conferred selective substrate recruitment in the same way that a corresponding E3 ligase ordinarily does for E2 ligases. This landmark report provided the foundation to creatively exploit the UPS for the benefit of human health, albeit by means of genetic engineering. The concept of using chimeric molecules capable of simultaneously recruiting a ubiquitin ligase and a target protein was first introduced with a patent filed by Proteinix [88]. However, the lack of specific biological data relating to target degradation left this report mostly unnoticed at the time of its writing in 2000. Separately in 2000, Zhou et al. succeeded in generating a recombinant E3 ligase from the SCF complex (skp-cullin-F-box) that is capable of recognizing the neosubstrate retinoblastoma protein (pRB) [89]. Collectively, these three reports represented important antecedents to the later harnessing of the UPS for TPD.

**Fig. 3:**
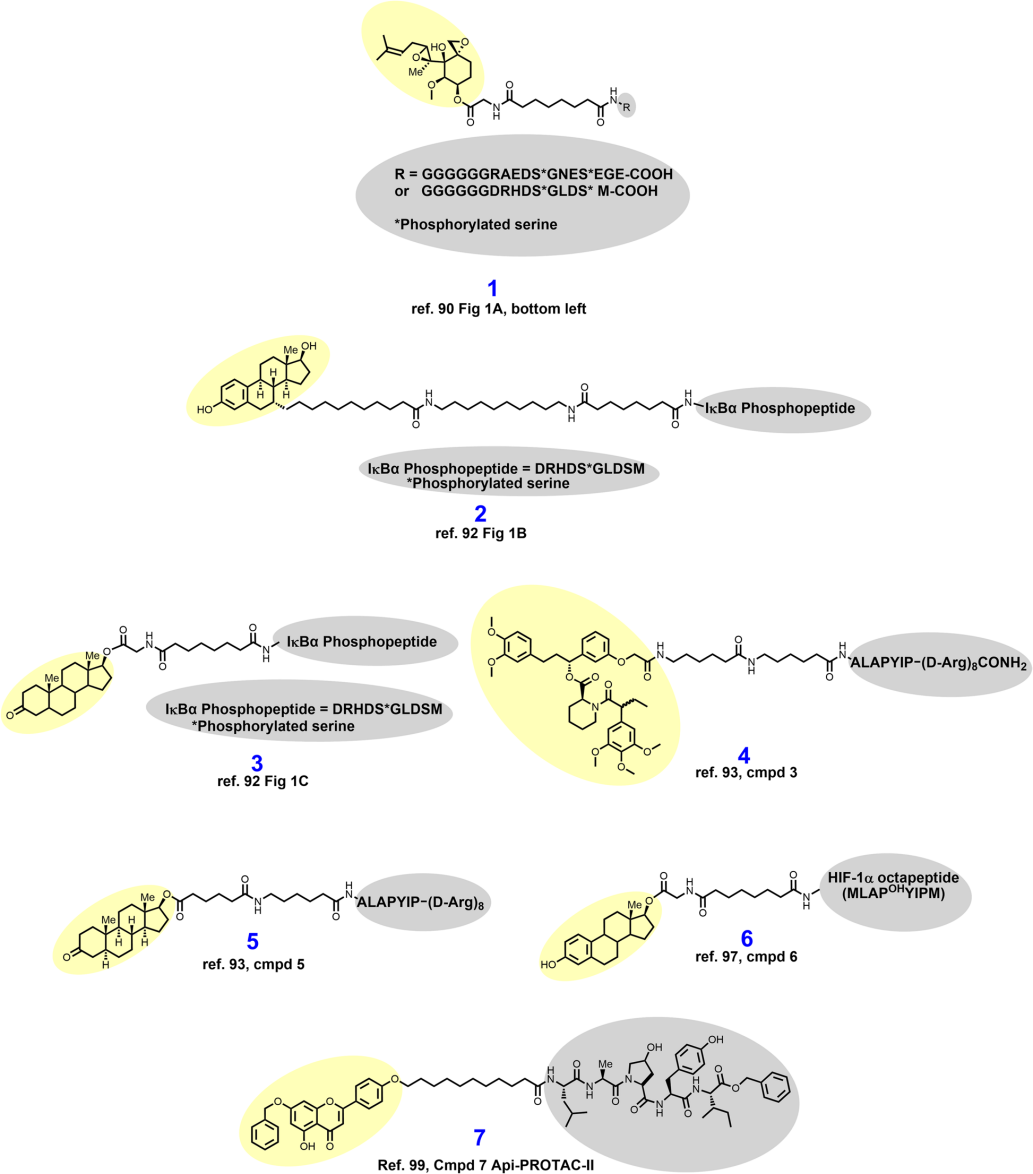
Early PROTACs employ peptidic E3 ligase ligands and lack cellular permeability. Targeting ligands and E3 ligase ligands are highlighted in yellow and gray, respectively

In 2001, Sakamato and colleagues from the laboratories of Raymond Deshaies and Craig Crews reported their discovery of aminopeptidase-2 (MetAP-2) degrader **1** (Fig. 3) based on the covalent ligand ovalicin [90]. Here, an eleven-atom alkylamide linker was used to append ovalicin to a peptide ligand for the E3 ligase SCFβ-TRCP [90, 91]. Sakamato et al. demonstrated that **1** recruits MetAP-2 in vitro in cellular lysates and results in ubiquitylation and proteasomal degradation of MetAP-2. Experiments could not be conducted in cellulo with **1** because of the poor cellular permeability conferred by the polypeptide SCFβ-TRCP ligand.

In the subsequent years, the laboratories of Crews & Deshaies sought to probe whether the PROTAC platform could be adapted to degrade other disease-associated proteins without using covalent warheads. To that end, in 2003 Sakamoto et al. reported the development of estrogen and androgen receptor degraders, **2** and **3**, respectively, by derivatizing estradiol and dihydrotestosterone [92]. Compounds **2** and **3** recruit SCFβ-TRCP by a ten AA IκBα-derived phosphopeptide appended by alkylamide linkers. With the cellular permeability issues of the PROTAC platform yet to be resolved, compound **2** was elucidated as an estrogen receptor (ER) degrader by in vitro incubation with purified ER and UPS members. Compound **3** delivered by microneedle injection was demonstrated to induce androgen receptor (AR) degradation by monitoring fluorescence in HEK 293 cells engineered to express GFP-AR.

In 2004, Schneekloth et al. reported their synthesis and biological assessment of the first cell-permeable PROTAC, **4**. PROTAC **4** is a VHL-dependent degrader of artificially generated FKBP12^F36V^ based on the F36V-specific ligand AP21998 and a VHL-recruiting minimal heptapeptide [93–95]. A recombinant target protein not expressed in the model cell line (HeLa cells) was selected here to demonstrate that cells could survive hijacking of the UPS and potential off-target degradation. The ALAPYIP VHL E3 ligase recruiting motif of **4** is capped by a polyarginine tail which was described elsewhere as a strategy for enhancing cellular permeability [96]. These target- and VHL-recruiting motifs are separated by an alkylamide linker in **4**. In recombinant HeLa cells, 25 μM **4** degrades GFP-FKBP12^F36V^ substantially, as monitored by fluorescence microscopy and Western blotting. Having shown that the design of cell permeable PROTACs is possible, Schneekloth et al. next sought to adapt this platform to target the AR because of its relevance in prostate cancer [93]. To this end, VHL-dependent AR degrader **5** was synthesized by replacement of AP21998 with testosterone and modifying the linker slightly (compare **4** to **5**). In recombinant HEK 293 cells, 25–100 μM **5** degrades GFP-AR, as detected by fluorescence microscopy and immunoblotting.

In 2004, Zhang et al. reported the cell permeable VHL-dependent ER degrader **6** which is structurally similar to **5** aside from utilizing the alternative HIF-1α octapeptide described elsewhere (compare **5** to **6**) [97, 98]. Both **6** and **5** use hydrophobic sterol-based targeting ligands to recruit the ER and AR, respectively, which likely improved cell permeability in these early PROTACs. In MCF-7 cells, **6** elicits ER degradation, although a 15-hour exposure at 100 μM was required to see maximal effects. VHL-dependence for ER degradation was demonstrated by reversibility of ER degradation upon mutation of the hydroxyproline in the HIF-1α octapeptide to alanine. Separately in 2007, Lee et al. reported VHL-dependent aryl hydrocarbon receptor (AHR) degrader **7** by derivatization of apigenin [99]. The AHR is a transcription factor that upon binding to certain environmental chemical carcinogens mediates carcinogenesis via epigenetic DNA damage [100–102]. The targeting ligand of **7** was modified by masking the 6-naphthyl hydroxyl group with a benzyl group because this hydroxyl group is unnecessary for AHR binding and poses metabolic liabilities [99]. The VHL-recruiting motif featured in compound **7** (H2N-LAP^OH^YI-benzyl ester) bears strong resemblance to VHL ligands seen in many modern PROTACs. In mouse hepatocytes immortalized by SV40 infection, 25–100 μM **7** induces degradation of the AHR. Orthogonally, recombinant CV-1 cells were engineered to express GFP-AHR, and in this model 10 μM **7** was observed to degrade GFP-AHR. PROTACs **1–7 **highjack the UPS for targeted protein degradation, but they nevertheless remained far from clinical candidacy because of high MWs, low potency, vulnerable peptide bonds, and, importantly, poor cell permeability.

## Nuclear hormone receptor degraders

### AR degraders

The AR is a 919 AA nuclear hormone receptor that is activated by testosterone or dihydrotestosterone (DHT) to mediate physiological processes related to the reproductive system among other functions [103, 104]. In prostate cancer, activation of the AR drives cancer growth and accordingly is the pharmacological target of clinically approved antiandrogens [3]. The mechanisms of antiandrogen resistance are complex but are thought to involve point mutations of the AR, formation of alternative splice products, and overexpression of the AR [13]. Moreover, continued AR signaling despite the presence of antagonists and/or depletion of androgens drives antiandrogen resistance in prostate cancer [25, 105–107]. PROTACs are iterative degraders that are more resilient to point mutations and therefore may be uniquely positioned to address antiandrogen resistance.

**Fig. 4:**
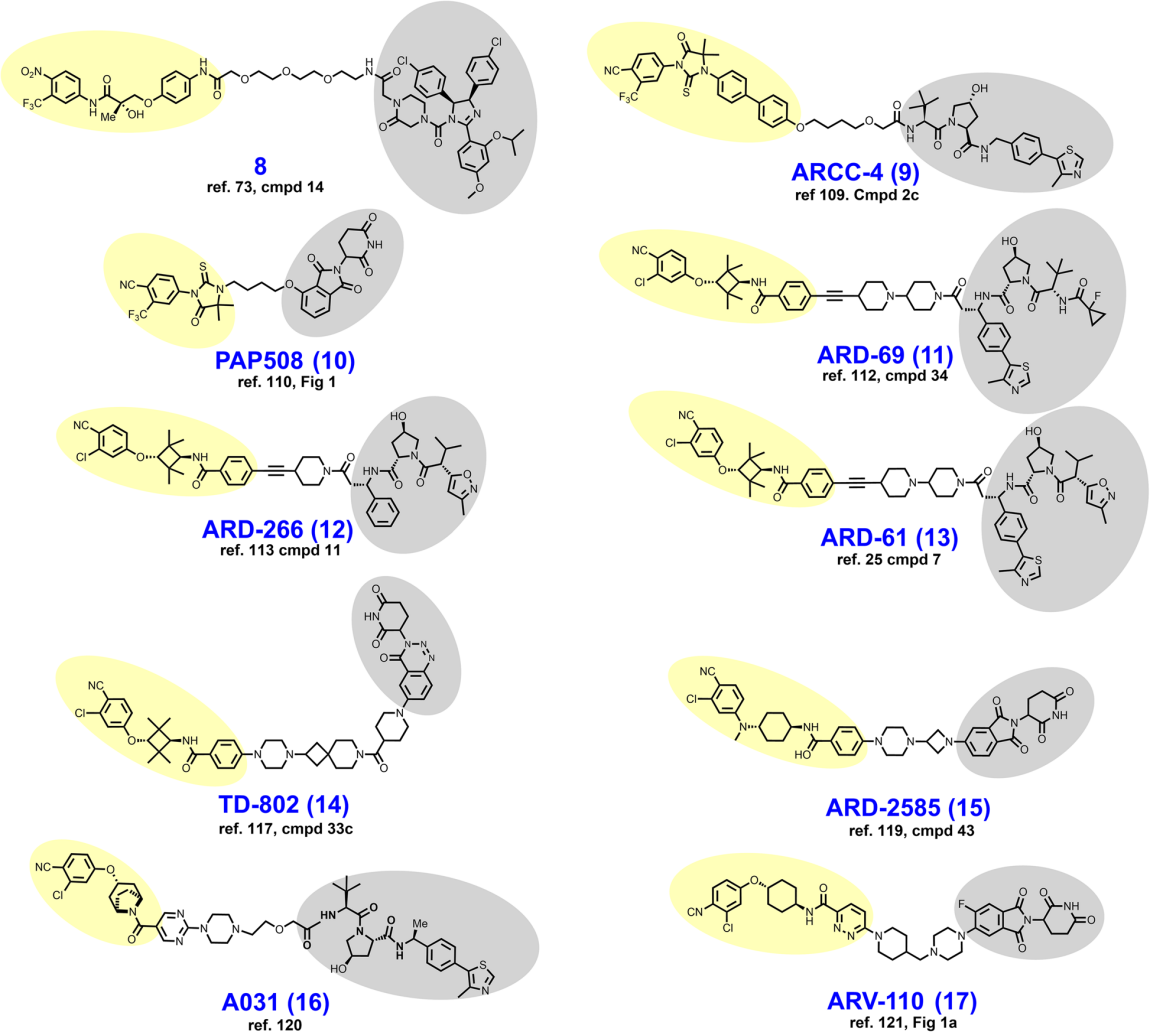
Structures of AR degraders. Targeting ligands and E3 ligase ligands are highlighted in yellow and gray, respectively

In 2008, Schneekloth et al. developed the first cell-permeable degrader **8** (Fig. 4), an AR-targeted PROTAC that discards the peptidic vestiges of the earlier PROTACs (compare **8 **to structures **1–7**) [73]. Here, the removal of peptidic vestiges was enabled by the discovery of imidazoline inhibitors of the E3-ligase Mdm2 [74]. Structurally, **8** employs this imidazoline ligand of Mdm2 as the E3-ligase recruiting element appended to a nitrobenzamide nanomolar inhibitor of the AR [108]. Unlike earlier PROTACs, **8** features a PEG linker. In HeLa cells ectopically expressing the AR, 10 μM **8** induces AR degradation which is reversible by proteasomal inhibition.

In 2018, Salami et al. reported their development of VHL-dependent AR degrader ARCC-4 (**9**) by bifunctionalizing enzalutamide with a hydrophobic ether linker [109]. In VCaP cells, ARCC-4 elicits a maximal degradation (D_Max_) of the AR exceeding 95% and half-maximal degradation (DC_50_) at 5 nM. ARCC-4 retains the ability to degrade the AR in recombinant HEK 293 cells engineered to express a panel of clinically relevant AR mutants. Selectivity for the AR was exhibited by no measurable loss of the paralogs ER, progesterone receptor, and glucocorticoid receptor on immunoblots at 1 μM.

In 2019, Da et al. reported their development of CRBN-dependent AR degrader PAP508 (**10**) which employs the hydantoin-based RU-59063 as the AR targeting ligand [110, 111]. RU-59063 and thalidomide were appended by a short four-carbon hydrophobic linker where thalidomide is attached by an ether linkage. As an AR-targeted PROTAC, PAP508 showed relatively modest AR degradation (D_Max_ < 50%) in both LNCaP and VCaP cell lines and required low micromolar concentrations to achieve maximal degradation. However, low micromolar concentrations of PAP508 produced favorable antiproliferative effects in viability assays, transwell invasion assays, and wound healing assays.

In 2019, Han et al. reported their medicinal chemistry campaign which culminated in the discovery of VHL-dependent AR degrader ARD-69 (**11**) [112]. In this report, the structural optimization efforts included the use of five distinct AR targeting motifs, a handful of different E3 ligase ligands targeting either VHL or CRBN, and numerous different linkers. Han et al. arrived at ARD-69 after determining that (i) a linker length corresponding to ~ 11 methylene groups was ideal, (ii) ethynyl attachment of an enzalutamide fragment outperforms amide attachment of the parent enzalutamide, (iii) heterocycles in the linker improve solubility, and (iv) that the VHL-ligand attachment is best achieved via a chiral position adjacent to the phenyl ring in the VHL-ligand (compare VHL ligand attachment in **11** to **9**, for example). In VCaP and LNCaP cell lines, ARD-69 induces nearly full depletion (D_Max_ > 95%) of the AR within 4 hours at a concentration of 100 nM and achieves a 24-hour DC_50_ of < 1 nM. In WST-8 cellular viability assays utilizing VCaP and LNCaP cells with growth stimulation by the AR agonist R1881, ARD-69 achieved IC_50_ values below 1 nM. In vivo, ARD-69 degrades tumor AR after a single intraperitoneal (IP) injection at 50 mg/kg given to mice xenografted with VCaP-derived tumors. Granted, tumor growth inhibition (TGI) was not reported here.

Han et al. published an additional paper in 2019 pertaining to the structurally similar ARD-266 (**12**). This report is noteworthy primarily because of the demonstration that AR-degradation was insensitive to VHL-ligand binding affinity over a wide range [113]. Structurally, ARD-266 differs from ARD-69 only by (i) containing one fewer piperidine rings in the linker, (ii) that the VHL-ligand is without the thiazole ring, and (iii) the cyclopropyl ring is replaced by a 3-methyl isoxazole ring (compare **11** to **12**). The modifications to the VHL-ligand in ARD-226 confer a loss of VHL-binding potency of nearly three logarithmic orders, yet both compounds perform similarly in the assays described in the previous paragraph. This study illustrates that in at least some chemical series PROTAC performance is relatively insensitive to binding affinity for the selected E3 ligase.

In 2020, Kregel et al. solidified the degrader rationale for the AR in prostate cancer from a mechanistic biology perspective [25]. The roles of alternative signaling pathways or activation of downstream binding partners independently of the AR in castration-resistant prostate cancer (CRPC) tumors remain controversial. AR splice variants such as AR-V7 pose a concern for resistance because the ligand-binding site is spliced out yet constitutive signaling continues [114, 115]. In this report, ARD-61 (**13**) was employed as a chemical tool to probe the molecular biology of enzalutamide-resistant cell lines and models overexpressing the AR splice variant AR-V7. To this end, Kregel et al. generated LNCaP recombinants ectopically overexpressing AR-V7 and assessed their sensitivity to ARD-61. To their surprise, the AR-V7 recombinants remained sensitive to ARD-61 in viability assays, nearly as much so as the parental LNCaP cell line. The notion that AR-V7 splice variants play an inconsequential role in tumor maintenance in CRPC was further substantiated using mice xenografted with CWR-R1 Enz^R^, a model whereby overexpression of AR-V7 has been validated [116]. In this xenograft model, tumor volume was approximately half that of control when ARD-61 was administered intraperitoneally throughout the study period.

In 2020, Takwale et al. reported their development of TD-802 (**14**), an AR-targeted PROTAC that is distinguished from other PROTACs through employing a novel benzotriazinone CRBN-recruiting motif that this group previously discovered [117, 118]. TD-802 employs the same tetramethylcyclobutane containing AR-recruiting element as the ARD series derivatives reviewed here (compare **14** to **11–13**). Structurally, the rigid linker in TD-802 includes a piperazine ring as an exit vector from the recruited AR, two piperidine rings, and a cyclobutane ring. Exploring various points of linker appendage to their novel CRBN ligand, Takwale et al. discovered that linker attachment via the 6th position provided the best AR degradation. TD-802 achieves a D_Max_ of 93% and DC_50_ at 12.5 nM in LNCaP cells. TD-802 was shown to be active in vivo when dosed IP to SCID mice bearing VCaP-derived tumors, albeit a TGI of < 50% was observed. Separately in 2021, Xiang et al. reported AR degrader ARD-2585 (**15**) which differs structurally from others in the ARD series in several ways (compare **15** to **11–13**) [119]. ARD-2585 is CRBN-dependent rather than VHL-dependent and discards the tetramethylcyclobutane ring of the targeting ligand, although an azetidine ring now appears in the linker. ARD-2585 is a picomolar degrader of AR^wt^ and degrades AR-V7, L702, and T878 variants in the picomolar - low nanomolar range. ARD-2585 is orally bioavailable and achieves better TGI than enzalutamide on a milligram: milligram basis in mice xenografted with VCaP cells. In 2021, Chen et al. reported the chemically dissimilar VHL-dependent AR degrader A031 (**16**), which utilizes an α-desmethyltropine-based targeting ligand [120]. In VCaP cells, A031 is a modest degrader of AR^wt^ at low micromolar concentrations. In zebrafish inoculated with fluorescently-labeled VCaP cells, A031 given aqueously at 8.3 μM displays TGI exceeding 50%.

Developed by Arvinas biopharmaceuticals, ARV-110 (**17**) is an orally bioavailable AR-targeted PROTAC that entered phase I/II clinical trials in March of 2019 where it is being assessed in metastatic CRPC (NCT03888612). ARV-110 appends its AR ligand to a CRBN-recruiting phthalimide via a rigid azaheterocycle-containing linker [121]. At the time of this writing, the drug development process and preclinical assessment of ARV-110 is yet to be published in a peer-reviewed journal. However, a presentation of this information was delivered at the 2021 annual AACR conference alongside a published abstract [122]. In this abstract, Snyder et al. report a DC_50_ of approximately 1 nM across a panel of prostate cancer cell lines and a D_Max_ exceeding 90% in in vivo models of prostate cancer. Snyder et al. furthermore presented proteomics data in support of favorable selectivity for the AR vs. other cellular proteins in the VCaP cell line, as well as robust tumor xenograft assay efficacy data. Earlier at the 2020 annual ASCO conference, interim clinical trial data were presented which suggested that ARV-110 is tolerable, although two of the twenty-two subjects experienced grade 3/4 elevations in liver enzymes, one of which subsequently developed acute renal failure [123, 124]. Arvinas attributes the observed hepatotoxicity to a drug interaction with the commonly utilized cholesterol lowering medication rosuvastatin which elevates the AST:ALT ratio as a single-agent.

### ER and estrogen-related receptor α (ERRα) degraders

In humans, the ER is a nuclear hormone receptor that spans ~475–600 AAs in length depending on which gene product (ERα or Erβ) and splice variant is produced [125]. Upon estrogen binding, the ER induces the transcription of gene products that control reproductive physiology among other functions. The current approach to the selection of treatment for breast cancer is determined by the presence or absence of various biomarkers and the histopathological grade among other considerations [104]. Estrogen-mediated activation of the ER has been implicated in both the development of and continued growth in ER+ breast cancer [2, 126]. Approximately 78% of breast cancer patients are ER+ at the time of diagnosis [127]. The initial pharmacotherapy of ER+ breast cancer commonly includes prescribing of an aromatase inhibitor, an ER antagonist, a selective ER modulator (SERM), or a CDK4/6 inhibitor [2]. Mechanisms of antiestrogen-resistance in ER+ breast cancer include conversion to ER- by loss of ER expression, mutations in the gene encoding the ER, altered expression of ER transcriptional coregulators, and compensation by other growth/survival pathways [14]. Although varied mechanisms have been described, there is evidence to suggest that continued ER signaling despite estrogen depletion or ER antagonism contributes to resistance [26]. Accordingly, the ER is a good candidate for a degrader-based approach. Fulvestrant is an FDA approved selective ER degrader (SERD) that induces ER degradation by disturbing its folding, nuclear localization, and homodimerization via a hydrophobic tail. However, fulvestrant is infrequently utilized because it requires parenteral administration and lacks efficacy in common ER mutants [128, 129].

Distinct from ERα and Erβ, a second pharmacological target highlighted in this section is the nuclear hormone receptor estrogen-related receptor α (ERRα). ERRα is thought to be an important mediator of tumor growth in ER- breast cancer given that (i) high expression of this target confers negative prognostic outcomes, (ii) loss of ERRα diminishes tumor growth, and (iii) there is demonstrable crosstalk between the pathways downstream of ER and ERRα [130]. ERRα has also been considered as a target for the treatment of type 2 diabetes given its role in promoting oxidative phosphorylation in muscle tissue, but it remains controversial whether an agonist/activator or an inhibitor/degrader is desired for this application [131–133]. There are no FDA approved modulators of ERRα.

**Fig. 5:**
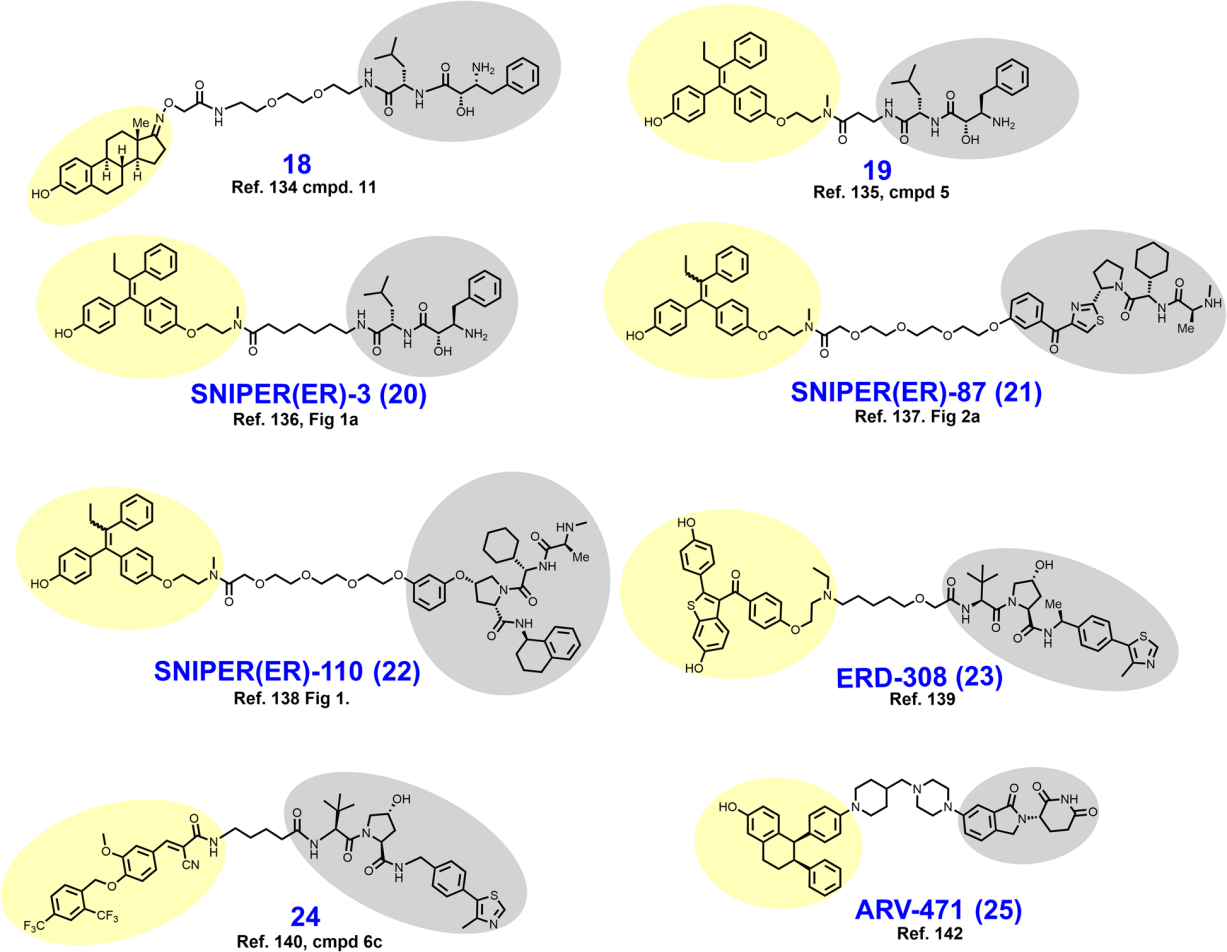
Structures of ER degraders. Targeting ligands and E3 ligase ligands are highlighted in yellow and gray, respectively

In 2011, Itoh et al. reported their development of cIAP1-dependent ER degrader **18** (Fig. 5) [134]. Here, **18** was conceived by ligation of the ER agonist estrone to an established ligand (BE04) of cIAP1 with a PEG-amide linker. Like its earlier counterpart **6**,  **18** appends the linker to the 17th position of the sterol ring structure but is distinguished by the use of an oxime group for linker attachment. In MCF-7 cells treated with 30 μM **18** for 24 hours, near complete degradation of the ER was observed. Following the report of **18**, additional cIAP1-dependent ER degraders **19–22** were reported which employ hydroxytamoxifen rather than estrone as targeting ligands [135–138].

In 2019, Hu et al. reported their development of VHL-dependent ER degrader ERD-308 (**23**), which employs a derivative of raloxifene as the targeting ligand [139]. This campaign began with replacement of the piperazine ring with a tertiary amine in order to gain a solvent exposed region for linker attachment. Their raloxifene derivative was then appended to either a CRBN ligand or a VHL ligand with varying linker lengths. Only the VHL-ligand bearing derivatives elicited ERα degradation below 1 μM in MCF-7 cells. Having now committed to recruitment of VHL as the selected E3 ligase, Hu et al. next determined an optimal linear linker length to be within the range of six to nine methylene units. Linker composition was best optimized by introducing the single oxyether unit present in ERD-308. In both MCF-7 and T47D cell lines, ERD-308 displays an impressive subnanomolar DC_50_ and achieves > 95% degradation below 5 nM.

In 2019, Peng et al. reported their discovery of compound **24**, an ERRα targeted PROTAC conceived by appending a VHL ligand to a fragment of a previously characterized inverse agonist (XCT790) of ERRα [140, 141]. The employed ERRα ligand was obtained by removal of the thiadiazole ring in XCT790, a modification that happened to improve binding affinity as detected by a TR-FRET assay monitoring the interaction of ERRα with PGC-1α coactivator. The acrylonitrile of the targeting ligand is suspected to bind covalently to Cys325 of ERRα based on in silico analysis. A four-carbon alkyl chain was used to append the ERRα ligand to the selected VHL ligand. In MDA-MB-231 cells, compound **24** elicits ERRα degradation with a DC_50_ in the low nanomolar range and achieves nearly full degradation at 100 nM. Notably, Peng et al. demonstrated that the PROTAC-induced loss of ERRα resulted in decreased expression of the downstream genes ATP5B, MCAD, and PDK4.

ARV-471 (**25**) is an orally bioavailable ER-targeted PROTAC developed by Arvinas that is currently being evaluated in Phase I/II clinical trials as monotherapy or in combination with the CDK4/6 inhibitor palbociclib for the treatment of ER+/HER2- metastatic breast cancer (NCT04072952). The linker of ARV-471 is structurally similar to the linker in the AR targeted PROTAC ARV-110 (compare **25** to **17**) that Arvinas advanced to clinical trials for the treatment of metastatic CRPC [142]. Both clinical candidates append their respective hormone receptor ligands to a CRBN-recruiting ligand via an azaheterocycle-containing linker that separates the piperazine and piperidine rings by a single methylene carbon. While the medicinal chemistry campaign arriving upon ARV-471 is yet to be published in a peer-reviewed journal, preclinical biological assessment data was presented at the 2018 San Antonio Breast Cancer Symposium [143]. There, Flanagan et al. described ARV-471 as an ER degrader with a DC_50_ of 2 nM that successfully reduces expression of ER-regulated genes and degrades clinically pertinent ER mutation variants. In both MCF-7 and patient-derived xenograft models, ARV-471 displays antitumor activity when dosed orally at 10 mg/kg. An interim clinical trial analysis (*n* = 12) was provided by Arvinas in December 2020. There, the clinical benefit rate associated with ARV-471 therapy was reported to be 42%, and no grade 3 or higher adverse events were observed [144].

## Kinase degraders

### Bruton’s tyrosine kinase (BTK) degraders

BTK is a 659 AA non-receptor tyrosine kinase that is expressed in most bone marrow-derived cell types including B cells where it is a known driver of hematological malignancies [15, 145]. BTK requires membrane-localization in order to activate pathways governing B cell growth and survival but exists in a cytosolic autoinhibited conformation until phosphatidylinositol-3,4,5-triphosphate (PIP3) levels rise [146, 147]. B cell receptor (BCR) activation is the impetus for PIP3 enrichment in the membrane to recruit BTK [145]. Membrane-associated BTK is activated via homodimerization and *trans*-autophosphorylation of the kinase domain [146, 147]. Oncogenic signaling events downstream of BTK include activation of PLCγ2, the MAPK pathway, NF-кB, and the AKT/mTOR signaling axis [145]. BTK is therefore a membrane-associated kinase that couples BCR activation to the activation of several pathways governing the growth and survival of B cells. Ibrutinib, acalabrutinib, and zanubrutinib are clinically approved BTK inhibitors approved for the treatment of B cell lymphomas. However, both primary and adaptive resistance mechanisms have been described including mutations to BTK, the BCR, and toll-like receptor (TLR) adaptor proteins [15]. Mutations to C481 in BTK are particularly concerning because this active site residue is the AA site targeted for covalent modification by the three available BTK inhibitors. The C481S mutation increases ibruitinib’s IC_50_ for inhibition of BTK^Y223^ phosphorylation from single digit nanomolar concentrations to ~ 1 μM [148]. As some binding affinity is retained for BTK^C481S^, ibrutinib and related BTK inhibitors may still prove useful as targeting ligands in the design of BTK degraders because target affinity is less stringent for PROTACs than occupancy-based inhibitors.

**Fig. 6:**
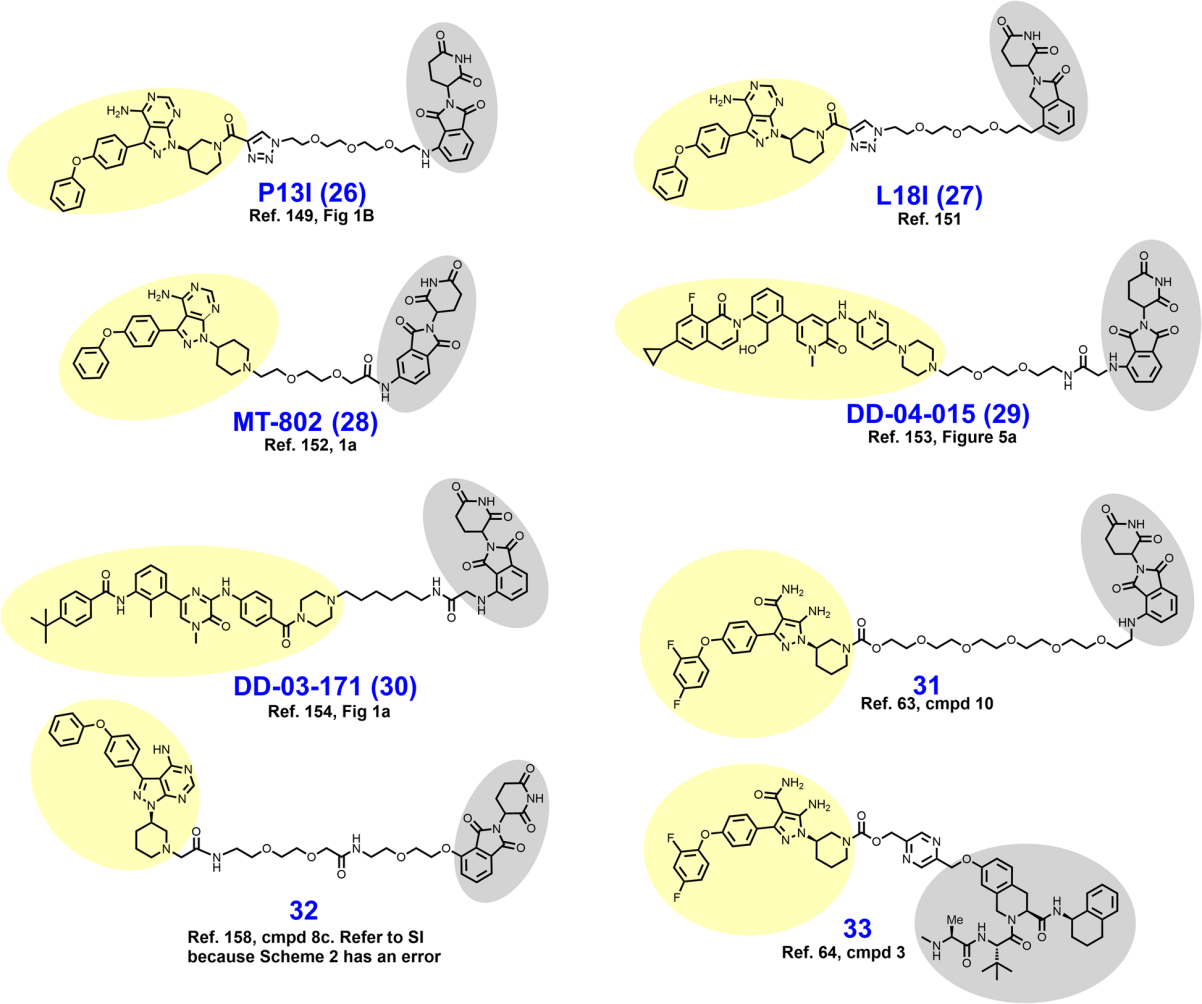
Structures of BTK Degraders. Targeting ligands and E3 ligase ligands are highlighted in yellow and gray, respectively

In 2018, Sun et al. reported P13I (compound **26** in Fig. 6), a BTK targeted PROTAC conceived by appending ibrutinib to a CRBN-recruiting ligand via a PEG-containing linker [149]. In this short chemical series, either pomalidomide or RG-7112 were employed to recruit E3 ligases CRBN or MDM2, respectively. Broadly, phthalimide-containing analogues outperformed their MDM2-recruiting counterparts. P13I degrades BTK with low nanomolar DC_50_ values in a variety of B cell lymphoma cell lines. Moreover, P13I degrades the clinically pertinent BTK^C481S^ mutant (DC_50_ = 30 nM) in recombinant HeLa cells. At 5 μM and 1 μM, respectively, P13I showed no off-target degradation and only minimal enzymatic inhibition of other kinases (ITK, EGFR, and TEC). Off-target binding of ITK, EGFR, and TEC are thought to mediate adverse events seen with clinical use of ibrutinib [150].

In 2019, Sun et al. reported L18I (**27**) as a culmination of their efforts to optimize P13I [151]. Goals of this campaign were to improve aqueous solubility, achieve in vivo activity, and degrade BTK mutants beyond C481S. Structurally, L18I differs from P13I by the replacement of pomalidomide with lenalidomide (compare **26** to **27**). This modification yielded a 4-fold improvement in aqueous solubility (L18I aqueous solubility = 8.6 mM) which enabled in vivo assessment. L18I achieves BTK^C481S^ degradation in HBL-1 cells in the low nanomolar range and outperforms ibrutinib in cellular proliferation assays using HBL-1, Mino, and Z138 cell lines. In mice xenografted with HBL-1 cells (BTK^C481S^), L18I demonstrates TGI when dosed IP at 30 or 100 mg/kg daily for 14 days.

In 2019, Buhimschi et al. described MT-802 (**28**), a CRBN-dependent BTK degrader that bears much structural resemblance to P13I (compare **26** to **28**) [152]. Early in the development of MT-802, Buhimschi et al. determined that attachment of the linker to the CRBN-recruiting phthalimide via C5 (rather than C4) allowed for shortening of the linker without loss of activity. VHL-ligand containing derivatives of MT-802 were synthesized in this series but yielded only modest BTK degradation. In Namalwa cells, MT-802 degrades BTK with a DC_50_ of 9.1 nM and achieves > 99% degradation at 250 nM. The targeting ligand found in MT-802 is a derivative of ibrutinib that discards the acrylamide and its covalent binding liability. Consistent with its reversible binding mode, MT-802 degrades BTK^C481S^ with similar potency as BTK^wt^.

While probing the degradable kinome in 2018, Huang et al. developed CRBN-recruiting BTK degrader DD-04-015 (**29**), which utilizes the reversible inhibitor RN486 as a BTK-recruiting ligand [153]. The linker composition is PEG-based and features a short acetamide bridge at the point of attachment to the isoindoline ring. In MOLM-14 cells, DD-04-015 degrades BTK in the low nanomolar range. In luciferase-based viability assays using TMD8 cells, DD-04-015 performed similarly to RN486. A year later in 2019 Dobrovolski et al. reported their development of lead compound DD-03-171 (**30**), a polydegrader of BTK, IKFZ1, and IKFZ3 [154]. The targeting ligand featured in DD-03-171 resembles RN486, although with subtle changes (compare **29** to **30**) in pursuit of BTK/IKFZ polydegradation. The linker of DD-03-171 features a hydrophobic linear alkyl chain instead of the repeating PEG unit seen in DD-04-015. DD-03-171 degrades BTK^wt^ with a DC_50_ of 5.1 nM and BTK^C481S^ in TMD8 recombinants with similar potency. In vivo, DD-03-171 given IP degrades BTK, reduces peripheral tumor cell counts, and prolongs survival in mice xenografted with DLBCL or MCL patient-derived samples.

In 2018, Zorba et al. sought to probe the role of cooperative PPIs between BTK and the selected E3 ligase in BTK degrader development [60, 63]. To this end, a library of eleven CRBN-dependent BTK targeted PROTACs containing PEG-based linkers ranging in length from three to nineteen atoms were synthesized. In this series, BTK was recruited through a covalently-binding arylpyrazole ligand and CRBN with a phthalimide appended to the linker at C4 (typified by **31**). The library of eleven BTK-targeted degraders were profiled for their capacity to form ternary complexes (BTK-PROTAC-CRBN) by FRET and SPR, and BTK degradation by immunoblotting. From this analysis, it was determined that cooperative PPI’s between BTK and CRBN were nonessential for potent BTK degradation while linker lengths of seven atoms or shorter promoted steric clashes which reduced degrader efficacy [63]. Proteomics studies revealed that their most potent BTK PROTAC **31** nonspecifically degrades the IMiD targets IKZF1, IKZF3, and ZFP91 [155–157].

In 2019, Krajcovicova et al. reported a CRBN-dependent nanomolar BTK degrader **32**, which employs a despropenoyl derivative of ibrutinib as the targeting ligand [158]. However, the most notable aspect of this report was their preparation of a thalidomide-preloaded resin (TPR) from aminomethyl polystyrene-divinylbenzene and subsequent application of this solid support system in degrader synthesis. Unlike traditional solution-phase synthesis, solid phase syntheses require less time and expertise for laborious isolation and purification steps. The scope of this report was limited to the synthesis of PROTACs employing kinase-targeting ligands with a solvent-exposed primary or secondary non-aromatic amine because conjugation was achieved through an elimination reaction via a 2-iodoacetamide in the TPR.

**Fig. 7:**
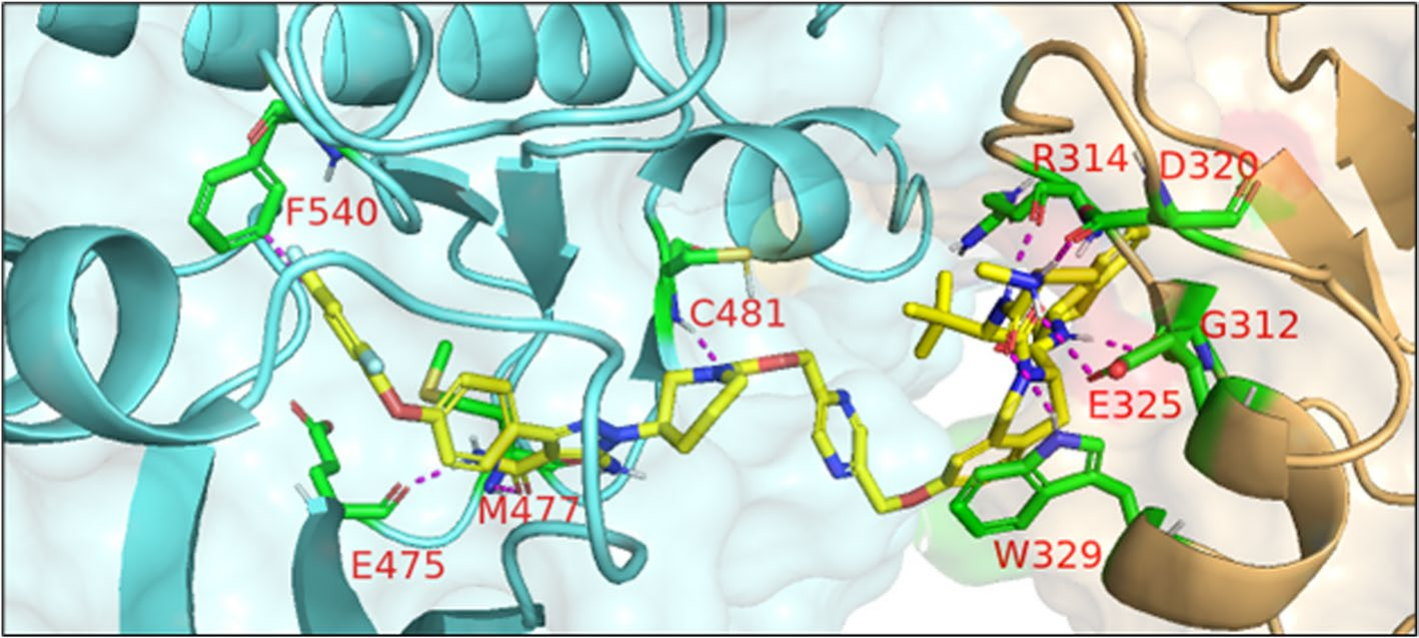
Crystal structure of cIAP1^Bir3^–compound **33**–BTK^KD^ ternary complex (PDB 6W7O). The cIAP1 ligand of **33** forms a π-cation interaction with cIAP1^R314^ and hydrogen bonds with cIAP1 residues G312, R314, D320, E325, and W329. The linker carbonyl interacts with BTK^C481^ through hydrogen bonding. The BTK ligand forms hydrogen bonds with BTK residues E475 and M477 while the difluorophenyl group π–π stacks with BTK^F540^. Image created using Schrödinger Bioluminate with PDB 6W7O. BTK^KD^ is depicted in cyan and cIAP1 in brownish orange

In 2021, Schiemer et al. solved the crystal structure of cIAP1-dependent BTK degrader **33** bound to cIAP1^Bir3^ and BTK^KD^ in a ternary complex (PDB 6W7O, illustrated in Fig. 7) [64]. The short pyrazine-containing linker of **33** was conceived by examining the ensemble of ternary complex poses displayed by a predecessor and directly bridging the BTK^KD^ and cIAP1^Bir3^ exit vectors. Shortening the linker in this way afforded a substantial reduction in MW and number of rotatable bonds, although a four-fold loss in BTK degrader potency was observed in THP-1 cells. Schiemer et al. furthermore elegantly demonstrated the formation of higher order cIAP1-PROTAC-target ternary complexes, providing a basis for PROTAC-induced cIAP1 autoubiquitylation.

### Anaplastic lymphoma kinase (ALK) degraders

ALK is a cell surface receptor tyrosine kinase (RTK) within the insulin receptor superfamily that under normal physiology is expressed in neuronal tissues and is most abundant during embryogenesis [159, 160]. In humans, the wt ALK gene encodes a 1620 AA class I membrane monospan [161]. The three tyrosine (Y1278, Y1282, and Y1283) kinase domain (AA 1116–1392) is found within the intracellular domain. After binding the extracellular ligand domain (AA 391–401), the endogenous ALK ligands pleiotrophin and midkine induce homodimerization and downstream signaling via PLCγ and the JAK-STAT, PI3K-Akt-mTOR, sonic hedgehog, and MAPK axes among others [160, 162, 163]. Expectedly, ALK is thus a prominent target in cancers where its dysregulation is linked to oncogenesis. ALK aberrations are frequently observed in anaplastic large cell lymphoma, NSCLC, and neuroblastoma among others [160]. The ALK gene found on chromosome 2 is particularly liable to gene translocations, and constitutively active/expressed oncogenic ALK fusion products have been described (i.e. NPM-ALK, EML4-ALK, etc) [160]. ALK amplification and mutations are also thought contribute to carcinogenesis in certain cancers [164–166]. Alectinib, brigatinib, lorlatinib, crizotinib, and ceritinib are inhibitors of the kinase domain that are FDA approved for ALK-positive tumors [167]. These occupancy-based inhibitors provide robust clinical responses that are limited only by the emergence of adaptive resistance mechanisms, the most prevalent of which are kinase domain point mutations [16].

**Fig. 8:**
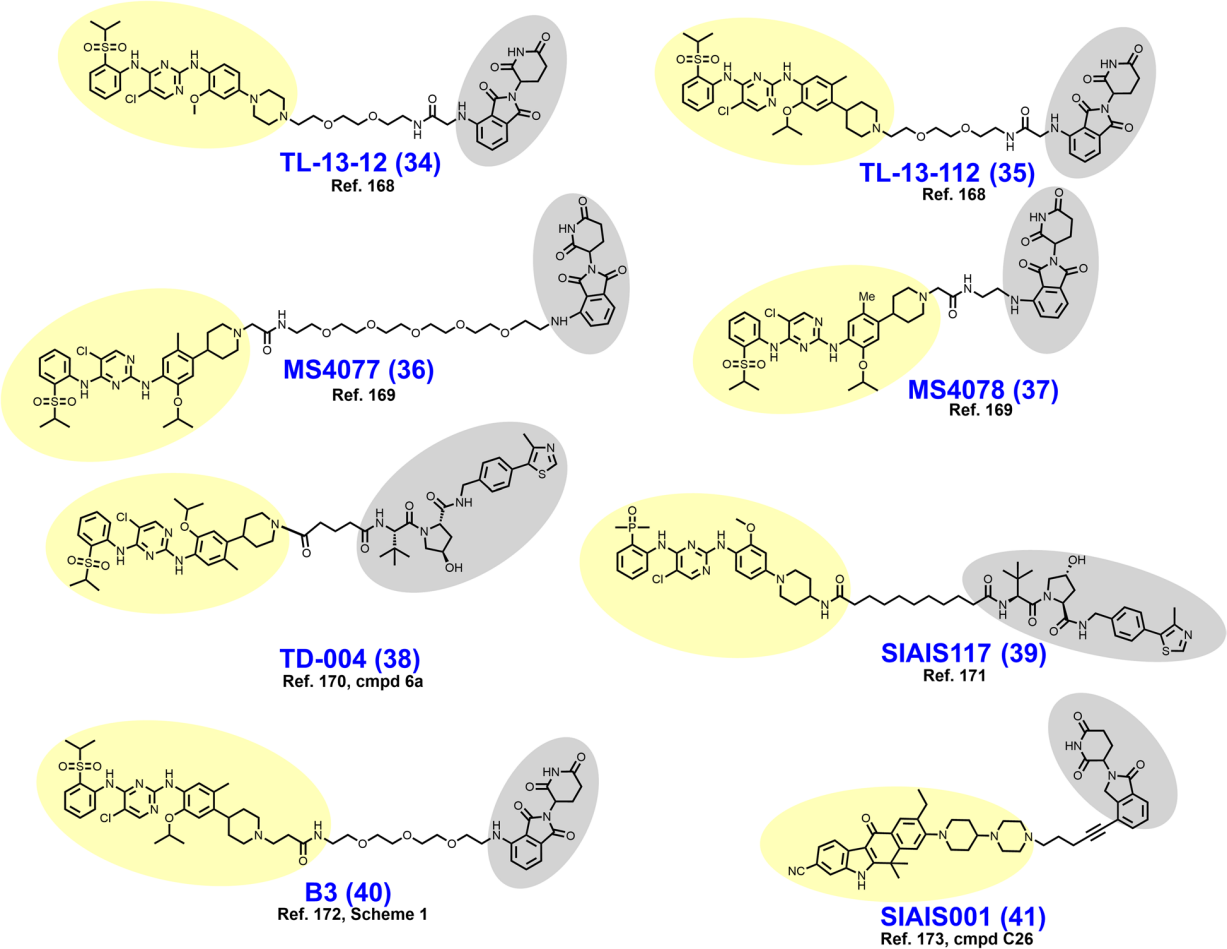
Structures of ALK degraders. Targeting ligands and E3 ligase ligands are highlighted in yellow and gray, respectively

In early 2018, Powell et al. reported the development of CRBN-dependent ALK degraders TL13–12 (**34**) and TL13–112 (**35**) (Fig. 8) [168]. TL13–12 and TL13–112 are PROTACs employing pyrimidine ALK inhibitors TAE684 and ceritinib, respectively, as targeting ligands. Depending on the ALK-addicted cell line assessed, Powell et al. observed that ALK degraders TL13–12 and TL13–112 performed more or less similarly to their respective parent reversible inhibitors in cell proliferation assays (low nanomolar EC_50_’s) and assessments of ALK and STAT3 phosphorylation. In H3122 cells, both ALK degraders achieve a D_Max_ > 99% with DC_50_ values of 10 nM, while potency was noticeably reduced in Karpas 299 cells. Collectively, TL13–12 and TL13–112 exhibited off-target degradation of targets commonly observed with kinase degraders including PTK2, FER, RPS6KA1, and Aurora A [153, 168]. Additionally, these researchers reported ABCB1 efflux as a potential liability for the PROTAC platform. Disappointingly, TL13–12 and TL13–112 performed similarly as the parent inhibitors in viability assays employing Ba/F3 cells engineered to express the clinically relevant EML4-ALK fusion mutants L1196M, C1156Y, and G1202R.

In a parallel pursuit, Zhang et al. reported their development of CRBN-dependent ALK degraders MS4077 (**36**) and MS4078 (**37**) based on linker derivatization of ceritinib with short acetamide- and PEG-based linkers, respectively [169]. ALK degraders MS4077 and MS4078 feature a 20–40 fold loss in binding affinity relative to ceritinib but exhibit DC_50_ values of 3 nM and 11 nM, respectively, in the NPM-ALK fusion bearing cell line SU-DHL-1. DC_50_ values in the mid two-digit nanomolar range were observed when tested with the EML4-ALK fusion containing NCI-H2228 cell line. An intriguing observation is that both MS4077 and MS4078 achieve potent ALK degradation despite their vastly different linker lengths. This observation suggests that distinct lysine residues are targeted for ubiquitylation or that the longer PEG linker assumes a higher order conformation resembling the rigid acetamide linker. A 50 mg/kg IP dose in mice provided an effective plasma concentration for 12 hours and an observed half-life exceeding 3 hours. No further in vivo data was reported for MS4077 and MS4078.

In 2018, Kang et al. reported the synthesis and biological evaluation of VHL-dependent ALK degrader TD-004 (**38**) [170]. TD-004 employs ceritinib as the ALK-targeting ligand and a linear alkyl chain to adjoin the two ligands. While TD-004 features amide attachment of the linker to the piperidinyl nitrogen of ceritinib, other analogues were synthesized that utilized tertiary alkyl amine-attachment. Amide-attached analogues achieved higher NPM-ALK degradation at 1 μM in SU-DHL-1 cells. In H3122 cells, TD-004 is a nanomolar degrader of the EML4-ALK fusion protein. Unlike previously reported ALK degraders, Kang et al. reported in vivo efficacy data. In mice xenografted with H3122 cells, TD-004 achieved tumor growth inhibition over 30 days when dosed IP daily at 58 mg/day.

In 2020, Sun et al. reported VHL-dependent ALK degrader SIAIS117 (**39**), which employs brigatinib as a targeting ligand after discarding the terminal piperazine ring and affixing a nine carbon diamidic alkyl chain [171]. PEG-amide or alkylamide linkers ranging in length from 3 to 118 atoms were evaluated in this campaign. SIAIS117 displays a DC_50_ of 7.0 nM in NPM-ALK fusion bearing SR cells and retains antiproliferative activity against HEK 293T cells engineered to express EML4-ALK^G1202R^.

In 2021, Yan et al. reported their development of CRBN-dependent ALK degrader B3 (**40**), a conception of linker derivatization of ceritinib with PEG chains [172]. Derivatization within their reported series of compounds focused on linker length (whether PEG or alkyl) and linker composition near the point of attachment of pomalidomide. In H3122 cells, B3 degrades nearly 100% of the EML4-ALK fusion protein in 8 h at 200 nM or within 24 h at 50 nM. EML4-ALK degradation correlated with a decrease in immunoblotting-detected downstream pSTAT3. While B3 exhibited off-target affinity for several nonselective targets of ceritinib, no appreciable degradation of these targets was observed up to 500 nM. In vivo, B3 given to rats at 1 mg/kg intravenously (IV) displays a half-life exceeding 4 h. In nude mice xenografted with H3122 cells, B3 achieved modest tumor growth inhibition at 25–50 mg/kg which was inferior to the parent ALK inhibitor ceritinib.

In 2021, Ren et al. reported the discovery of CRBN-dependent ALK degrader SIAIS001 (**41**) after linker-iMiD derivatization of an alectinib analogue [173]. As VHL-dependent analogues of an initial screen library were less potent than the CRBN-dependent counterparts, phthalimides were appended to various linkers for the remainder of the campaign. SIAIS001 appends its respective ligands by a short pentyne linker. In NPM-ALK fusion-bearing SR cells, SIAIS001 degrades ALK with a DC_50_ of 3.9 nM and a D_Max_ of 70.3%.

### Fms-like tyrosine kinase 3 (FLT3) degraders

FLT3 is an RTK that drives the development of acute myeloid leukemia (AML) and is closely related to other hematopoietic RTKs including PDGFR, M-CSFR, and c-KIT [174, 175]. Under normal physiology, the FLT3 encoding gene is principally expressed in hematopoietic stem cells and progenitor cells where it supports replenishment of differentiated bloods cells. Full length human FLT3 is 993 AAs in length and is trafficked to the membrane as a monospan (C-terminus, cytosolic) by an N-terminal signal recognition peptide (AA 1–26) [175, 176]. Extracellular dimeric FTL3 ligand induces receptor homodimerization/oligomerization to produce a conformational change in the juxtamembrane (JM) domain (AA 594–610), allowing ATP loading of the kinase domains (AA 611–993) [175, 177]. Following ATP-loading, reciprocal autophosphorylation of the intracellular kinase domains proceeds and produces downstream signaling via the PI3K/AKT and MAPK pathways which control cell growth and survival. Common cancer-associated FLT3 mutations include (i) internal tandem duplications (ITDs) within the JM domain that disrupt autoinhibition of the kinase domain; and (ii) activating mutations (i.e., D835X) in the activation loop of the kinase domain [174, 178]. First-line therapy for AML is typically the multikinase inhibitor midostaurin in combination with “7 + 3” (cytarabine cycled with an anthracycline), which provided an overall survival of 75 months in the RATIFY trial [179, 180]. Additional FLT3/multikinase inhibitors include sorafenib, quizartinib, crenolanib, and gilteritinib [17]. Primary and secondary resistance mechanisms to FLT3 inhibition have recently been described [17].

**Fig. 9:**
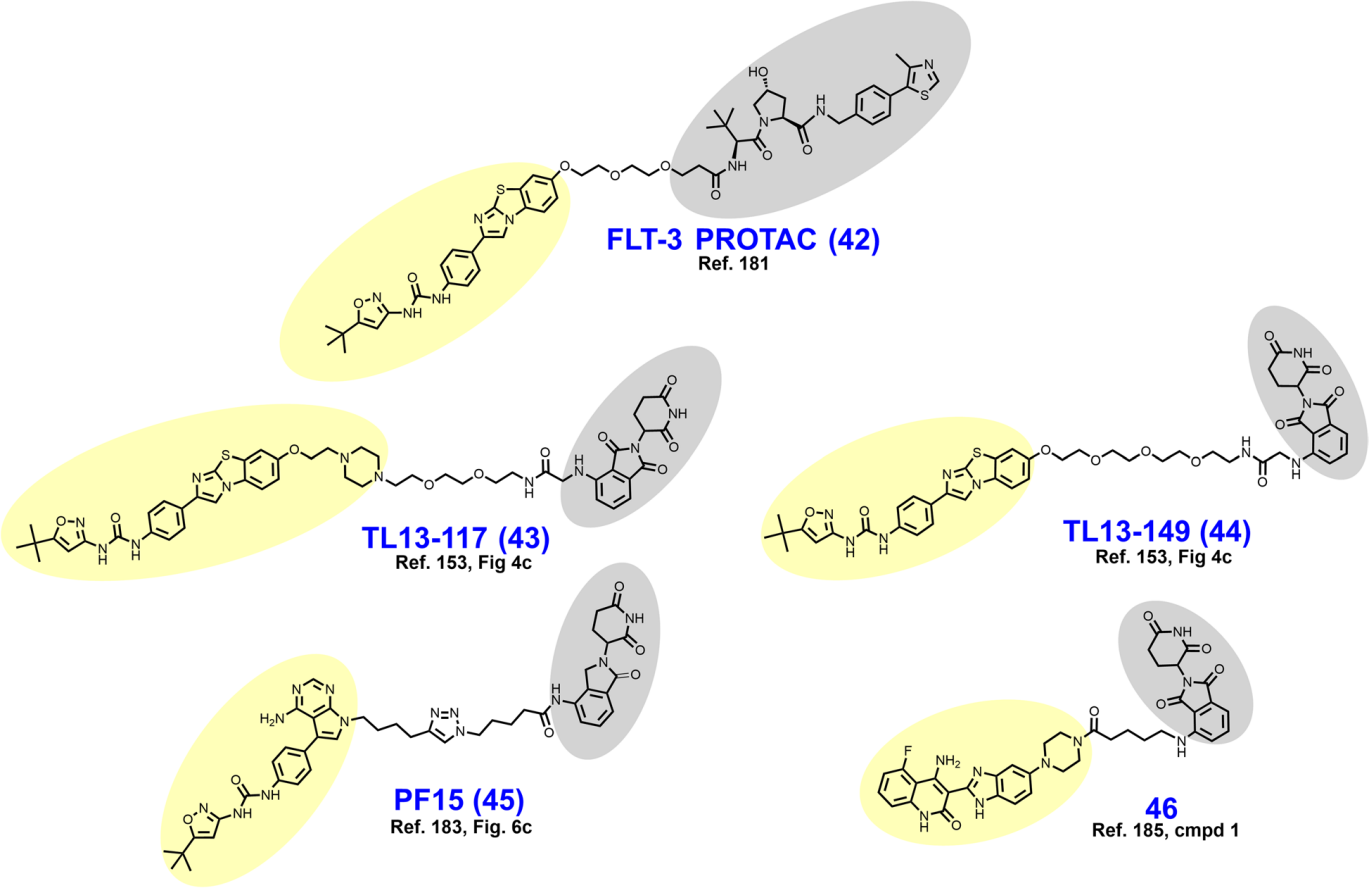
Structures of FLT3 degraders. Targeting ligands and E3 ligase ligands are highlighted in yellow and gray, respectively

In 2018, Burslem et al. reported VHL-dependent FLT3 degrader FLT-3 PROTAC (**42**) (Fig. 9) based on removal of the morpholine ring of quizartinib and growing a PEG-chain in its place [181]. Compared to quizartinib, FLT-3 PROTAC engages fewer targets at 1 μM in the Kinome scan assay, and in proteomics studies no targets other than FLT-3 were appreciably depleted. In MV4–11 and MOLM-14 cells, FLT3-PROTAC degrades FLT3 in the low nanomolar range and retains antiproliferative activity against MOLM-14 cells engineered to express a second clinically relevant mutation (D835Y or F691L) beyond ITDs. In vivo, 30 mg/kg IP given daily for 3 days reduced FLT-3 levels by ~ 60% in nude mice xenografted with MV4–11 cells.

As a part of a larger query of the degradable kinome, Huang et al. reported TL13–117 (**43**) and TL13–149 (**44**) as CRBN-dependent FLT3-selective degraders in 2018 [153]. The clinical candidate quizartinib, a selective, competitive inhibitor of ATP-binding to FLT3, was utilized as the targeting ligand for these compounds [182]. Structurally, both TL13–117 and TL13–149 feature a PEG-based linker, although TL13–117 replaces one PEG unit with a piperazine ring (compare **43** to **44**). Demonstration of a UPS-dependent mechanism of degradation for these degraders was complicated by that (i) FLT3 is constitutively degraded by autophagy and (ii) occupancy-based FLT3 inhibition induces FLT3 upregulation [153]. TL13–117 and TL13–149 exhibit FLT3 degradation in the range of 10–100 nM which is not reversible by autophagy inhibition, and which is subject to a hook effect at higher concentrations. In antiproliferative assays employing MOLM-14 and MV4–11 cells, however, the parent inhibitor AC220 achieved 5-fold lower IC_50_ values.

In 2022, Chen et al. reported the development of CRBN-dependent FLT3 degrader PF15 (**45**) which employs an analogue of a pyrrolopyrimidine FLT3 inhibitor as a targeting ligand [183, 184]. Structurally, PF15 features a thirteen-atom alkyl-based linker with a triazole ring situated midway. Derivatization in this campaign focused on modulation of the alkyl chain length on either side of the triazole. Bifunctionalization of the parent inhibitor dramatically improved selectivity for FLT-3 relative to c-KIT, an off-target kinase whose inhibition is associated with bone marrow suppression [183]. In FLT3-ITD bearing BaF3 cells, PF15 degrades nearly 100% of FLT3 in 24 h at a concentration of 100 nM and displays a DC_50_ of 76.7 nM. In vivo, PF15 given at 10 mg/kg IP exhibits a TGI of 58.4% in mice bearing BaF3 tumors. In 2021, Cao et al. reported the dovitinib-based CBRN-dependent degrader **46** which degrades FLT-3 in the single-digit nanomolar range, although c-KIT was also substantially depleted in this range [185].

### Cyclin-dependent kinase 9 (CDK9) degraders

CDK9 is a ubiquitously expressed serine/threonine (S/T) kinase belonging to a group of noncanonical cyclin-dependent kinases (CDKs) that govern transcription rather than cell cycle progression [186]. CDK9 is 372 AAs in length and contains domains broadly conserved across kinases including other CDK isoforms [186]. Gene transcription is typically regulated at the step of elongation beyond 20–50 mRNA bases by factors that induce pausing of RNA polymerase II [186]. The cyclin-CDK9 complex phosphorylates these factors to allow gene transcription to resume. Heightened CDK9 activity has been linked to development or maintenance of numerous hematological and solid malignancies because CDK9 promotes continuous expression of MCL-1 and c-myc [186]. CDK9 is also thought to be an ATR- and Rad3-binding partner involved in the replication stress response and maintenance of genomic stability [187, 188]. While several CDK4/6 inhibitors have received FDA approval and a variety of other pan-CDK and isoform selective inhibitors are in development, no specific CDK9 inhibitors are approved for clinical use [189].

**Fig. 10:**
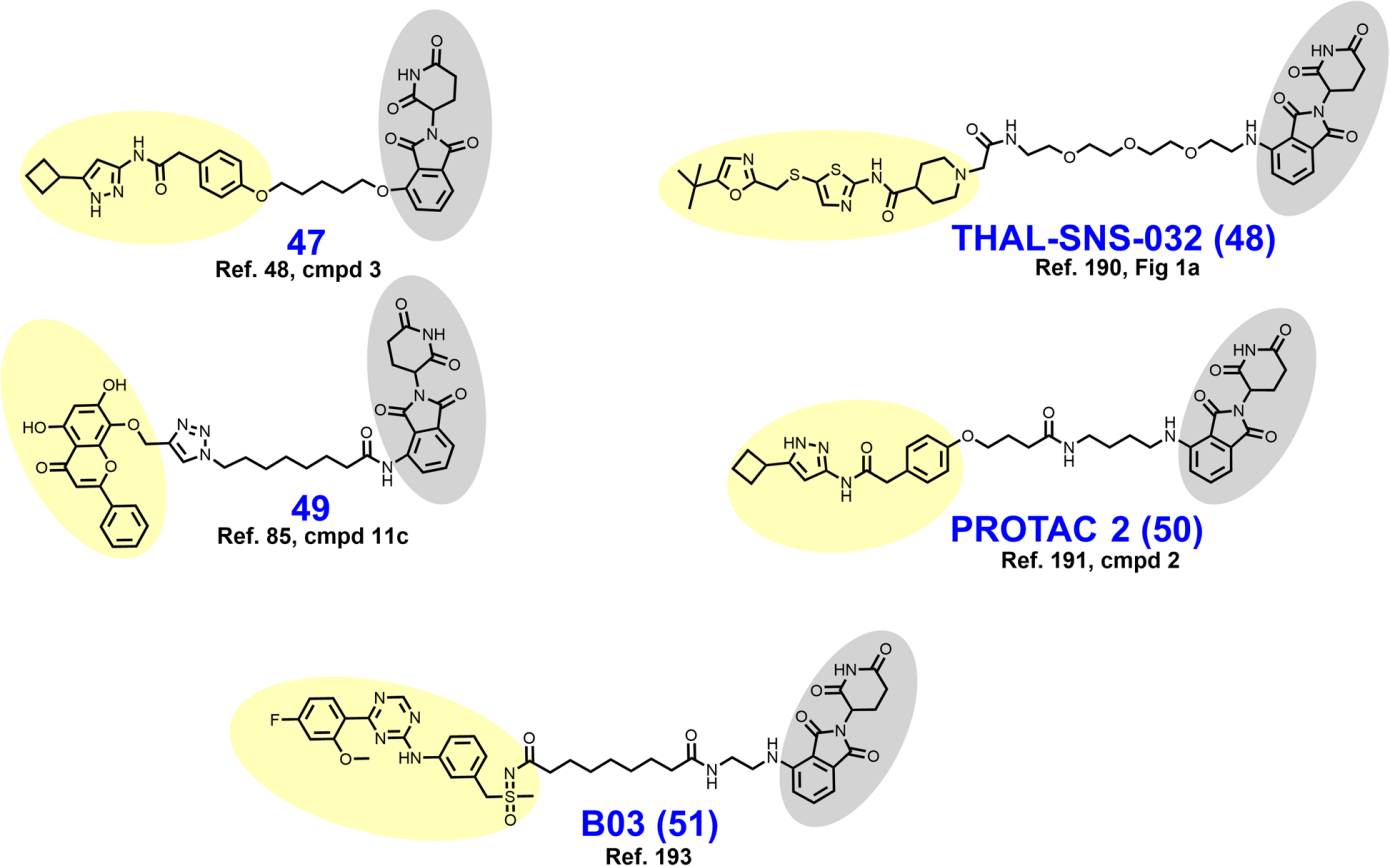
Structures of CDK9 degraders. Targeting ligands and E3 ligase ligands are highlighted in yellow and gray, respectively

In 2017, Robb et al. reported CRBN-dependent CDK9-selective degrader **47** (Fig. 10) after derivatizing an aminopyrazole targeting ligand with a short alkyl linker [47, 48]. In HCT116 cells, 20 μM **47** depletes more than 60% of CDK9 with no detectable changes in CDK2/5 levels. These researchers attributed the improved selectivity of **47** vs. its parent inhibitor as a consequence of the differential surface lysine residue patterning on CDK9 compared to CDK5. Mechanistically, the depletion of CDK9 induced by **47** correlates with a reduction in downstream phosphorylated RNA Pol II and MCL-1 expression.

In 2018, Olson et al. reported the development of CRBN-dependent CDK9 degrader THAL-SNS-032 (**48**) [190]. PEG-imide modification of the parent multikinase inhibitor SNS-032 provided a mid-nanomolar range degrader of CDK9 that was CDK isoform selective, albeit partial degradation of CDK10 was observed. An orthogonal tandem mass tag proteomics screen confirmed that CDK9 was the most substantially depleted target out of 4512 proteins assessed and the only CDK isoform displaying greater than a 2-log-fold reduction. In parallel, Olsen et al. attempted to generate CDK9 degraders using the highly selective CDK9 warhead NVP-2 but were unable to obtain an efficacious CDK9 degrader by this route. THAL-SNS-032 achieved similar reductions in mRNA expression and RNA Pol II mislocalization as the selective CDK9 inhibitor NVP-2.

In 2018, Bian et al. reported CRBN-dependent CDK9 degrader **49** which employs the natural product wogonin as a targeting ligand and a triazole-containing linker [85]. The triazole within the linker arises from a “clickable” azido-pomalidomide designed by Bian et al. to improve the throughput of their syntheses. Excitingly, the triazole-containing PROTACs demonstrated improved CDK9 degradation compared to non-triazole wogonin-PROTACs also reported here. **49** degrades CDK9 in the low double-digit micromolar range in MCF-7 cells treated for 24 hours. No alterations in the levels of CDK2/4/5/7/8 were observed. **49** outperformed the parent CDK9 inhibitor wogonin in MCF-7 antiproliferative assays with an IC_50_ of 17 μM and induced apoptosis, as determined by flow cytometry.

In 2021, King et al. reported CRBN-dependent PROTAC 2 (**50**) which retains the same aminopyrazole targeting ligand as **47** and is a direct successor [191]. Comparatively, PROTAC 2 features (i) an increase in the linker length from seven to eleven atoms, (ii) an additional carboxamide in the linker, and (iii) replacement of the ether-linked isoindoline for pomalidomide (compare **47** to **50**). PROTAC 2 achieves a DC_50_ of 158 nM compared to their previous micromolar degrader **47**. Similar to **47**, PROTAC 2 retains binding affinity and antikinase activity for other CDK isoforms yet does not degrade these targets *in cellulo*. In a resazurin-based viability assay employing MIA PaCa-2 cells, PROTAC 2 exhibits strong synergy with FDA approved Bcl2 inhibitor venetoclax. A resistance mechanism to Bcl2 inhibitors mediated by MCL-1 (governed in turn by CDK9) has previously been described [192].

In 2021, Qiu et al. reported the development of CRBN-dependent CDK9 degrader BO3 (**51**) after derivatizing the triazine selective CDK9 inhibitor BAY-1143572 [193, 194]. Structurally, BO3 appends pomalidomide by an amidic alkyl chain. BO3 achieves 100% degradation of CDK9 within 6 h in MV4–11 and MOLM13 cells at a concentration of 500 nM and a DC_50_ of 7.62 nM. In MV4–11 cells, CDK9 degradation by BO3 occurs within 1 h and renders undetectable MCL-1 levels within 12 h. After a single 5 mg/kg IV dose administered to mice bearing MV4–11 xenografts, BO3 reduces tumor CDK9 levels by approximately 50%.

### Focal adhesion kinase (FAK) degraders

Broadly, FAK (also known as PTK2) is a protein involved in cell adhesion, motility, growth, and survival [195]. FAK is a 1052 AA non-receptor tyrosine kinase that when cytosolic assumes an autoinhibited conformation whereby the F2 domain obstructs access to the kinase domain (AA 411–686) [196]. FAK also contains a C-terminal focal adhesion targeting domain which interacts with integrin adapter proteins to enable FAK dimerization, attachment to cytoskeletal proteins, focal adhesion complex formation, Y397 autophosphorylation, and initiation of Src-FAK signaling [195]. Signaling by the dual kinase Src-FAK complex promotes metastatic invasion, anchorage independent growth, and angiogenesis in cancer [197]. While there are no FDA approved FAK inhibitors, BI-853520/IN-10018, defactinib, CEP-37440, GSK2256098, and PF-00562271 are clinical stage FAK inhibitors +/− dual kinase inhibitory activity under evaluation for various metastatic cancers [198–206]. The pertinent nonenzymatic roles of this kinase in focal adhesion complex assembly make FAK a particularly suitable target for a degrader [24].

**Fig. 11:**
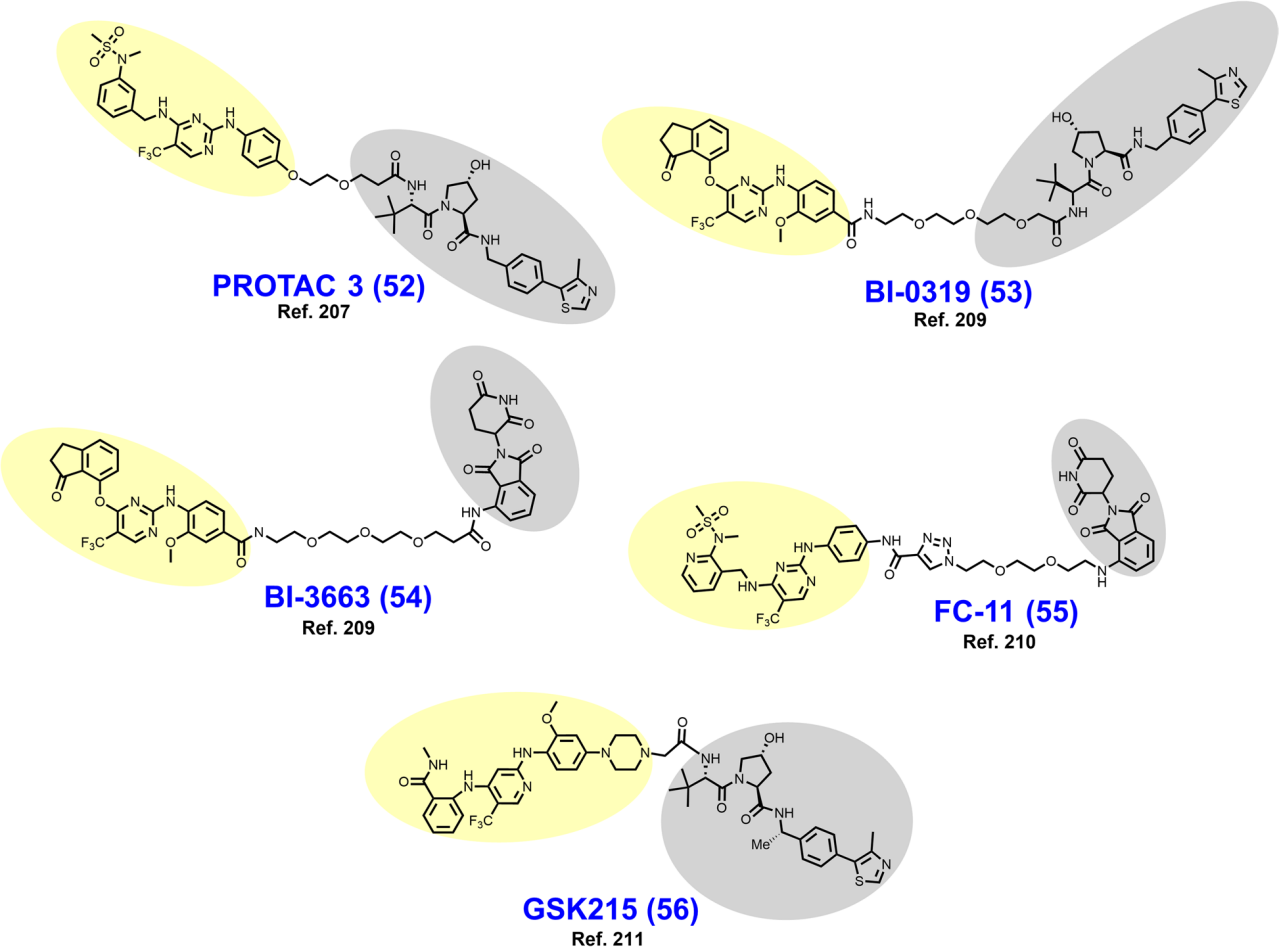
Structures of FAK degraders. Targeting ligands and E3 ligase ligands are highlighted in yellow and gray, respectively

In 2018, Cromm et al. reported the VHL-dependent FAK degrader PROTAC 3 (**52**) (Fig. 11) composed of a defactinib-like warhead and a short ether-based linker [207]. The *N*-methylbenzamide of defactinib was discarded to allow for direct ether attachment of the linker to the phenyl ring, a strategy to reduce peptidic character and improve cellular permeability. PROTAC 3 is substantially more selective for FAK-binding vs. other kinases compared to defactinib. In PC3 cells treated for 24 hours, PROTAC 3 degrades > 99% of FAK with a DC_50_ of 3 nM. In this series, the linker length and composition SDRs were astringent, as a variety of linkers afforded potent FAK degradation. At equipotent doses in MDA-MB-231 and PC3 cells, respectively, PROTAC 3 more effectively prevents cell migration and phosphorylation of downstream FAK substrates than defactinib. The favorable in vivo activity of PROTAC 3 was subsequently explored in KRAS mutated NSCLC models by Liu et al in a detailed 2021 report [208].

In 2019, Popow et al. reported FAK degraders BI-0319 (**53**) and BI-3663 (**54**) [209]. Both degraders employ FAK inhibitor BI-4464 as a targeting ligand, PEG-based linkers, and differ only in their selection of E3 ligase ligand (compare **53** to **54**). In A549 cells treated for 18 h, both degraders exhibit half-maximal degradation of FAK at ~ 7 nM, although CRBN-recruiting BI-3663 exhibits a higher D_Max_ of 95%. BI-3663 and BI-0319 feature exquisite selectivity for FAK as determined by a Kinome Scan (off-target binding) and proteomics (off-target degradation). Both PROTACs exhibit low permeability which was attributable to drug efflux by P-glycoprotein on the basis that cyclosporine A rescues FAK degradation.

In 2020, Gao et al. reported the CRBN-dependent femtomolar FAK degrader FC-11 (**55**) (DC_50_ = 80 fM) featuring a triazole-containing PEG-based linker [210]. The targeting ligand in FC-11 is similar to although distinct from the pyrimidine-based FAK ligands found in PROTAC 3, BI-3663, and BI-0319 (compare **55** to **52–54**). Here, thirty-nine chimeric analogues were described which employ a variety of triazole-containing diethylene- and triethylene-glycol linkers. Within this campaign, shorter linkers displayed the most robust FAK degradation in the PA1 cells employed as a primary screen. Additionally, amine, alkyl, and alkyne groups were explored as differential modes of phthalimide attachment. Alkyne attachment was shown to hinder FAK degradation. Furthermore, the orientation of the amide between the FAK ligand and the triazole in FC-11 was noted to be of importance. Like other reported FAK degraders, FC-11 did not exhibit markedly improved antiproliferative effects beyond the parent inhibitor.

**Fig. 12:**
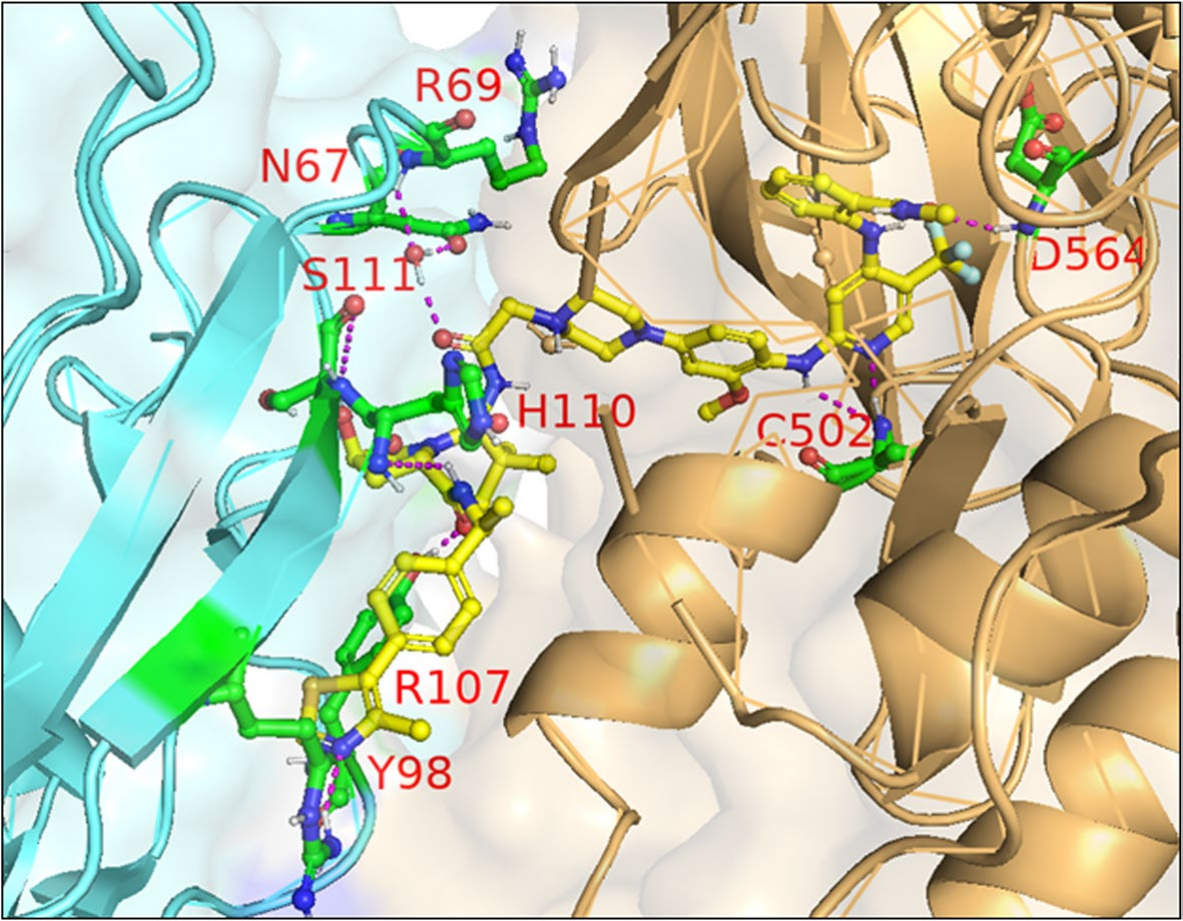
Crystal structure of the VHL-ElonginC-ElonginB (VCB)-GSK215-FAK ternary complex (PDB 7PI4). The VHL-FAK neo-PPI interface maps to 2661 Å^2^, the largest of any reported PROTAC ternary complex. The VHL ligand is buried within a pocket defined by both the surface of VHL and FAK. Hydrogen bonding interactions with VHL residues Y98, R107, H110, and S111 are shown. The linker carbonyl forms water-mediated hydrogen bonds with VHL residues N67 and R69. Additional hydrogen bonds with FAK residues C502 and D564 are shown. Image created using Schrödinger Bioluminate with PDB 7PI4. VHL is depicted in cyan and FAK in brownish orange

In 2021, Law et al. discovered the VHL-dependent FAK degrader GSK215 (**56**) by derivatizing a previously reported 2,4-diaminopyridine FAK inhibitor [211, 212]. Therein, alkyl and PEG-based linkers ranging in length between two and fourteen atoms were explored. Broadly, shorter linkers in this series afforded more potent FAK degradation with GSK215 featuring the two atom acetamide linker and displaying single digit nanomolar FAK DC_50_ values in A549 cells. Law et al. furthermore solved the crystal structure of the extraordinarily cooperative (α > 100, by FRET) VHL-GSK215-FAK ternary complex (PDB 7PI4, illustrated in Fig. 12). Multiplexed proteome dynamics profiling revealed a distinct set of degraded off-targets relative to other FAK degraders including all members of the CDK-activating kinase complex. In vivo, GSK215 degrades approximately 85% of hepatic FAK in CD1 mice when given as a single SQ injection at 8 mg/kg.

### Breakpoint cluster region protein (BCR) and Abelson’s tyrosine kinase (ABL) (BCR-ABL) fusion protein degraders

The BCR-ABL fusion protein is a historically important target arising from the Philadelphia Chromosome, the first identified genetic link to cancer [213]. Chronic myeloid leukemia (CML) is characterized by a reciprocal translocation between chromosomes 9 and 22 that produces a gene fusion product on chromosome 22 between BCR and ABL. The translocation typically exchanges a region of exon 1 of BCR with the N-terminal cap region of ABL rendering a kinase no longer able to assume its autoinhibited conformation [214, 215]. In wt nonreceptor tyrosine kinase ABL, the SH2 and SH3 domains bind the C-lobe of the kinase domain, obstructing access to the active site until activation by Y245/Y412 phosphorylation or binding by SH2/SH3 domain containing proteins [216, 217]. Constitutively active BCR-ABL signals through downstream pathways or effectors including MAPK, PI3K-Akt, STAT5, and CrkL to promote carcinogenesis [218]. FDA approved occupancy-based BCR-ABL inhibitors include (i) first generation imatinib which only binds when the activation loop is in the closed (inactive) position; (ii) second generation dasatinib, nilotinib, and bosutinib which bind irrespective of the activation loop position; (iii) third generation ponatinib which remains active against the T315I mutation; and (iv) allosteric (myristoyl-binding regulatory pocket) inhibitor asciminib [219, 220]. Resistance to BCR-ABL inhibition is typically due to point mutations in the P-loop or ATP-binding pocket, gene amplification, drug efflux, or BCR-ABL-independent mechanisms [18].

**Fig. 13:**
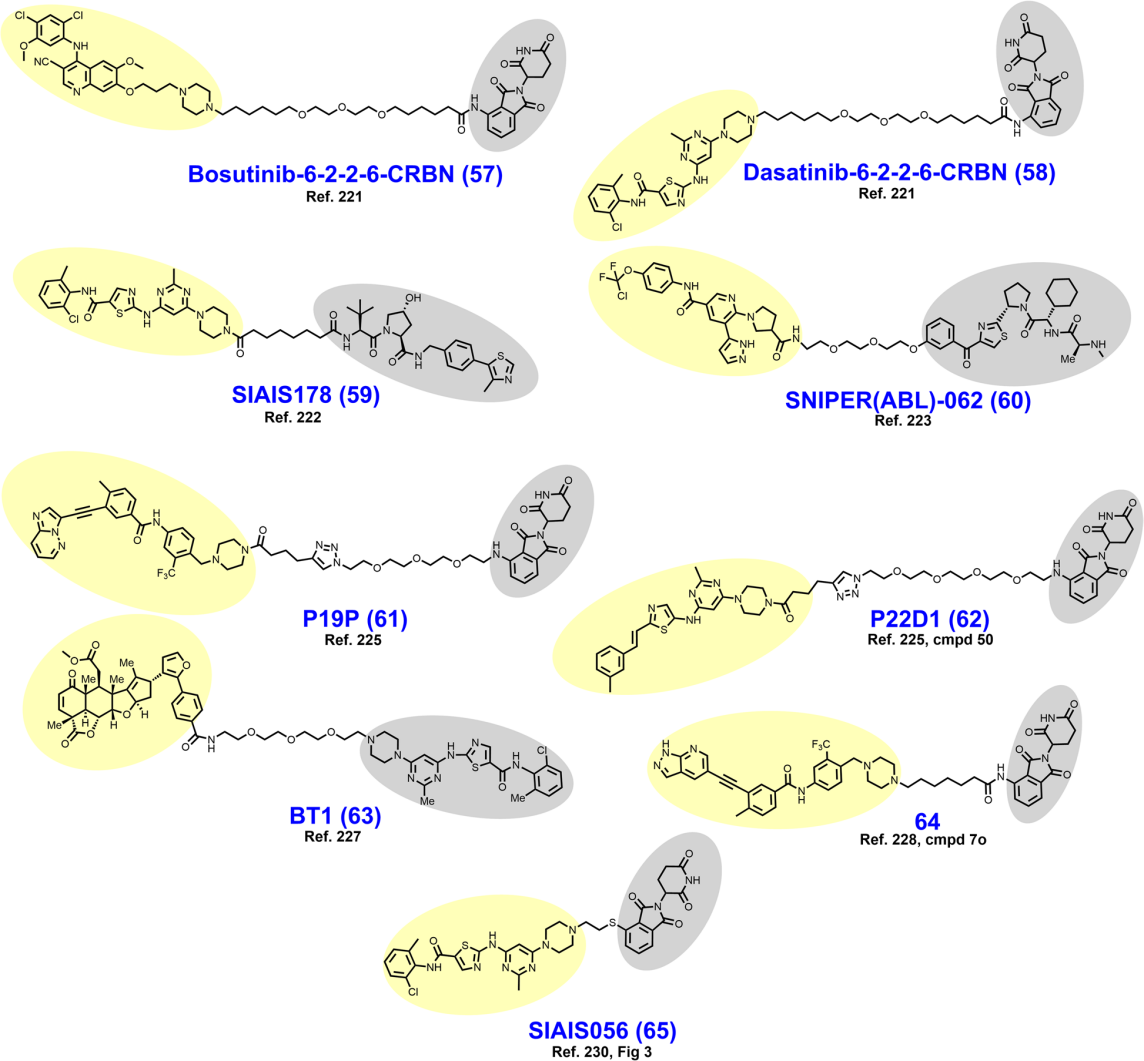
Structures of BCR-ABL degraders. Targeting ligands and E3 ligase ligands are highlighted in yellow and gray, respectively

In 2016, Lai et al. published a report suggesting that BCR-ABL may be differentially susceptible to CRBN-mediated degradation compared to VHL ligase. Therein, a series of BCR-ABL targeted chimeras derivatized based on tyrosine kinase inhibitor (TKI) warhead, E3 ligase ligand, and linker properties were described [221]. All PROTACs synthesized displayed a loss of ABL affinity relative to the parent TKI, although the losses were greatest among PROTACs with hydrophobic linkers. The authors attributed this finding to the propensity of hydrophobic linkers to collapse and impose an entropic barrier to binding. Lai et al. treated K562 cells for 24 hours followed by immunoblotting as their primary screen for identifying BCR-ABL/ABL degraders. None of the compounds utilizing imatinib displayed BCR-ABL/ABL degradation. Similarly, none of the chimeras employing a VHL ligand achieved BCR-ABL degradation, although several were noted to be ABL degraders. However, chimeras utilizing a CRBN ligand displayed dual-degradation of BCR-ABL/ABL if bosutinib or dasatinib was employed as a warhead. The structures of Bosutinib-6-2-2-6-CRBN (**57**) and Dasatinib-6-2-2-6-CRBN (**58**), micromolar and nanomolar degraders of BCR-ABL, respectively, are shown in Fig. 13.

In contrast to the above report, Zhao et al. described their success in degrading BCR-ABL through VHL-recruiting PROTACs in a 2019 report culminating in the development of SIAIS178 (**59**) (DC_50_ = 8.5 nM) [222]. Observing that the best BCR-ABL degraders reported by Lai et al. employed dasatinib as the targeting motif, this TKI was selected as the targeting ligand. Ultimately, Zhao et al. were able to convert the dasatinib-VHL ligand chimeras reported earlier into active degraders by (i) shortening the linker to 5–10 atoms, (ii) exchanging ether-composed linkers for alkyl chains, and (iii) utilizing an amide-attachment at the piperazine ring. SIAIS178 induced formation of stable ternary complexes with VHL and BCR-ABL, as determined by size exclusion chromatography, pull-down assays, and the nanobit platform. Tandem mass tag proteomics determined that the degrader profile of SIAIS178 was far more selective than its binding profile would predict. SIAIS178 induced degradation of BCR-ABL mutants in recombinant U937 cells, although several including T315 mutants were non-degradative targets. SIAIS178 attenuates K562 tumor growth in xenografted mice, although the parent TKI dasatinib remained the most efficacious in this assay.

In 2017, Shimokawa et al. reported cIAP1-dependent BCR-ABL degrader SNIPER (ABL)-062 (**60**) based on the allosteric inhibitor asciminib [223]. In K562 cells treated for 6 hours, SNIPER (ABL)-062 exhibits a D_Max_ of ~ 70% and a DC_50_ of ~ 30 nM. Similar to other cIAP1-dependent degraders where cIAP1 autoubiquitylation has been observed, SNIPER (ABL)-062 induced degradation of cIAP1 which may limit its efficacy [75, 223, 224]. Treatment of K562 cells with 100 nM SNIPER (ABL)-062 reduced the levels of downstream pSTAT5 and CrkL to a similar degree as asciminib at an equimolar concentration.

In 2020, Yang et al. reported their efforts to broaden the chemical toolbox available for developing BCR-ABL degraders. In this pursuit, the researchers reported their development of CRBN-dependent BCR-ABL degraders P19P (**61**) (DC_50_ = 20 nM) and P22D1 (**62**) [225]. Previous attempts to develop a BCR-ABL degrader employing an orthosteric ligand (i.e., ponatinib, imatinib, nilotinib) had failed. Compared to other such attempts, Yang et al. attained a ponatinib-based degrader P19P through utilizing a much longer triazole-containing PEG-based linker [225, 226]. A practical computational method for analyzing ternary complex dynamics is described by Yang et al. which supports their findings related to orthosteric BCR-ABL degraders. Also noteworthy within this report is the modification of dasatinib by exchanging the chlorobenzamide with a methylstryrene to enable affinity for BCR-ABL^T315I^. This dasatinib derivative was subsequently utilized as a warhead in degrader P22D1 which degrades BCR-ABL^T315I^ at 1 μM in recombinant Ba/F3 cells. Several other BCR-ABL mutants were successfully degraded by analogues reported here. Separately in 2020, Tong et al. reported their success in expanding the toolbox of E3 ligase ligands for BCR-ABL degradation [227]. Here, the researchers reported RNF114-dependent BCR-ABL degrader BT1 (**63**), which preferentially degrades BCR-ABL relative to ABL, albeit at low micromolar concentrations.

In 2021, Jiang et al. reported CRBN-dependent BCR-ABL^T315I^ degrader **64** based on their clinical candidate 3rd generation BCR-ABL inhibitor olverembatinib [228, 229]. Jiang et al. began by appending pomalidomide, VHL ligand, or a cIAP1 ligand to olverembatinib by PEG linkers [228]. Using Ba/F3 cells engineered to express BCR-ABL^T315I^, an initial screen among PEG linker PROTACs was performed, but only the amide-attached CRBN-dependent derivative achieved appreciable degradation at 300 nM. Satisfied with their selection of a CRBN-ligand, derivatives were synthesized employing either PEG- or alkyl-based linkers of various lengths. While many of the derivatives afforded modest BCR-ABL^T315I^ degradation, only **64** (corresponding to a 6-carbon alkyl linker) achieved > 90% degradation at 300 nM. Degrader **64** retains the capacity to degrade BCR-ABL^wt^ in K562 cells, although several of the earlier, less potent derivatives did not induce measurable degradation in this model. **64** displays single-digit nanomolar IC_50_ values in viability assays employing K562 or Ba/F3 cells. In vivo, 20 mg/kg **64** given IP every other day induces tumor growth inhibition > 90% in mice xenografted with recombinant Ba/F3 cells engineered to overexpress BCR-ABL^T315I^.

In 2021, Liu et al. reported CRBN-dependent BCR-ABL degrader SIAIS056 (**65**) after derivatizing dasatinib with a short ethyl linker and a sulfur-attached iMiD [230]. Early in their series of CRBN-recruiting BCR-ABL degraders, Liu et al. determined that both alkyl- and PEG-based linkers of various lengths yielded low nanomolar DC_50_ values for BCR-ABL in K562 cells. Having identified that linker length is not a decisive factor, a series of PROTACs containing three atom linkers were synthesized with the intent of restricting molecular weight. Among these analogues, it became apparent that sulfur attachment of the iMiD affords improved BCR-ABL degradation compared to *N-*, alkyl-, or *O-* attached iMiDs. The last SDR elucidated in this report is that alkyl attachment of the linker to the piperazine of dasatinib elicits more potent BCR-ABL degradation than when the linker is *N-*acylated. SIAIS056 retains the capacity to degrade a handful of BCR-ABL mutants associated with TKI resistance, although the T315I variant remained resistant. In mice xenografted with K562 cells, SIAIS056 outperforms dasatinib on a milligram: milligram basis and achieves full tumor regression when given at 10 mg/kg/day.

### Epidermal growth factor receptor (EGFR) degraders

EGFR is a member of the ErbB family which includes other cell surface RTKs such as HER2, HER3, and HER4 [231]. Structurally, EGFR is a 1186 AA membrane monospan with an N-terminal extracellular domain (AA 1–621), a transmembrane domain (AA 622–644), and intracellular domains (AA 646–1186) including the JM, SH1, and C-terminal tail domains [231]. EGF and TGF-α represent high affinity EGFR ligands that bind the extracellular ligand-binding site to induce a conformational change, enabling EGFR homo/heterodimerization [232]. Dimerization of RTKs enables autophosphorylation of the c-terminal tail domains and subsequent recruitment of adaptor proteins (i.e., Shc, Grb proteins, SHP proteins) for initiation of downstream oncogenic signaling via the MAPK, PI3K-AKT, and JAK-STAT pathways [231]. Often coinciding with receptor amplification, EGFR sequence aberrations are common in cancer and confer growth factor-independent kinase signaling and resistance to receptor endocytosis [19, 233]. The most common activating mutation in EGFR is L858R. FDA approved 1st and 2nd generation EGFR inhibitors include gefitinib, erlotinib, afatinib, and dacomitinib, all of which inevitably succumb to resistance by T790M or other adaptive resistance mechanisms. Third-generation EGFR inhibitors osimertinib and vandetanib provide coverage for the T790M mutation but remain vulnerable to site C797 mutations among other acquired resistance mechanisms [19]. Several monoclonal antibody therapies are available which bind to the extracellular domain of EGFR to prevent growth factor binding and receptor dimerization, although their efficacy is limited [234].

**Fig. 14:**
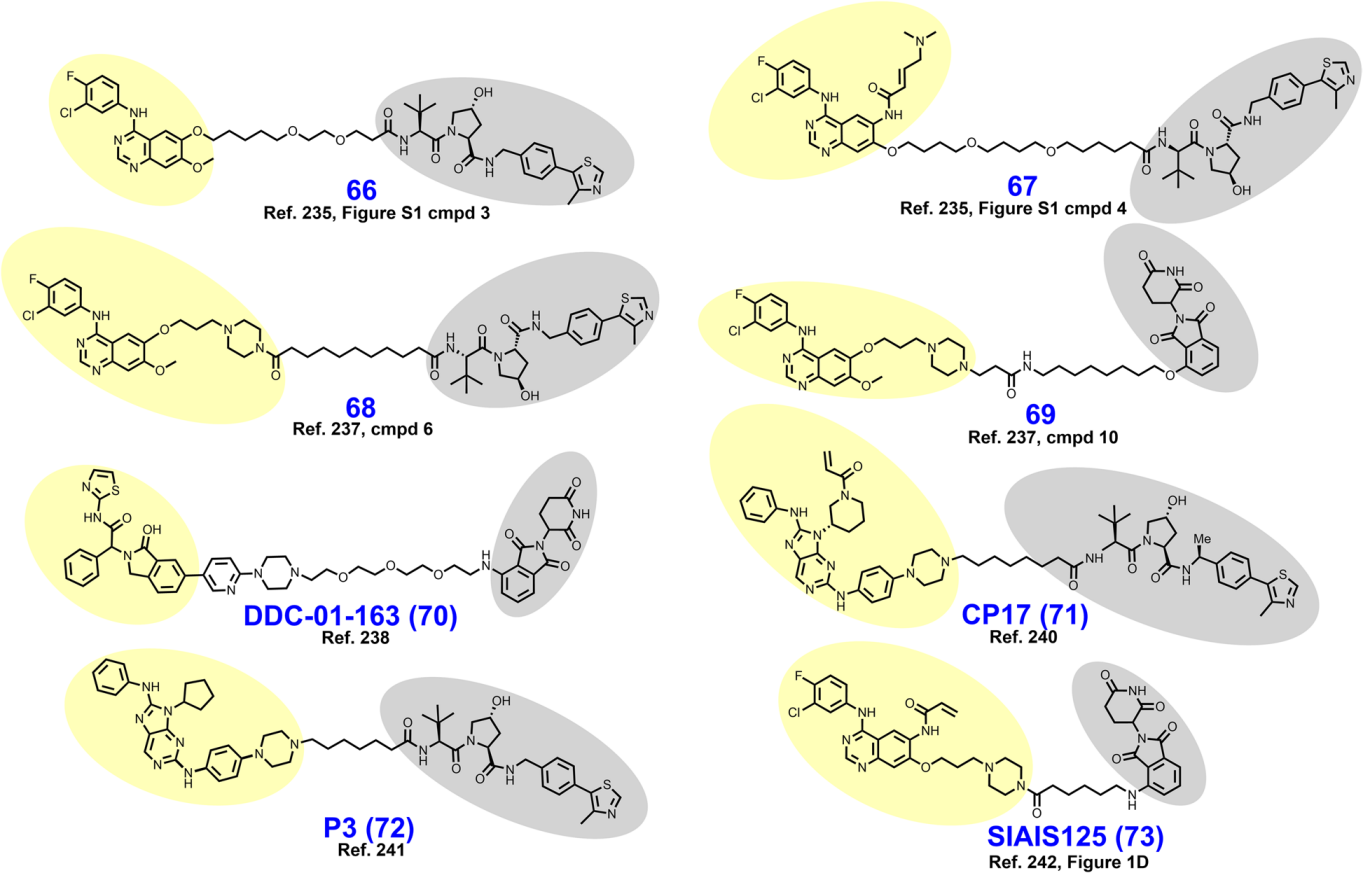
Structures of EGFR degraders. Targeting ligands and E3 ligase ligands are highlighted in yellow and gray, respectively

In 2018, Burslem et al. published a proof-of-concept pertaining to the development of RTK targeted PROTACs [235]. Therein, the mutant-selective TKI gefitinib was utilized to develop VHL-dependent mid-nanomolar range degrader **66** (Fig. 14) of EGFR^del19^ and EGFR^L858R^ which spares EGFR^wt^. Here, an afatinib-based degrader **67** featuring epimerization of the tertbutyl substituent was also reported to degrade the double-mutant EGFR^L858R/T790M^ at micromolar concentrations. Of importance, the degrader rationale for RTKs was cemented in this report by establishing that EGFR degradation prevents kinome rewiring by receptor heterodimerization, a liability associated with occupancy-based inhibition [235, 236].

In 2020, Cheng et al. reported VHL-dependent EGFR degrader **68** and CRBN-dependent EGFR degrader **69**, which both employ a piperazine-containing analogue of gefitinib as the targeting ligand [237]. Irrespective of E3 ligase ligand, longer alkyl chain linkers provided more robust degradation than PEG-based and short alkyl linker analogues. In HCC-827 (EGFR^del19^) and H3255 (EGFR^L858R^) cells, degraders **68** and **69** deplete over 95% of the respective targets below 50 nM with DC_50_ values in the single-digit to low double-digit nanomolar range. Cheng et al. observed that serum starvation, as in the tumor microenvironment, enhances EGFR degradation, a finding the authors speculated is related to enhanced receptor endocytosis. Both **68** and **69** are selective degraders of EGFR^del19^ and EGFR^L858R^ relative to EGFR^wt^, an unexpected finding since **69** displays higher binding affinity for EGFR^wt^ than the mutation variants. Both **68** and **69** were demonstrated to be bioavailable after IP administration to mice, although the half-life of the VHL-ligand bearing **68** was substantially longer.

In 2020, Jang et al. reported CRBN-dependent EGFR degrader DDC-01-163 (**70**) which is distinguished by utilizing an allosteric site-binding isoindolinone targeting ligand reported earlier [238, 239]. Because of the unique EGFR binding mode, DDC-01-163 retains the ability to degrade EGFR variants (i.e., C797X and L718X) that are associated with resistance to 3rd generation TKIs. The linker SDRs within this series were fairly stringent and required a 3-PEG unit and attachment to the phthalimide at C4.

In 2022, Zhao et al. reported the development of the covalent EGFR degrader CP17 (**71**) which employs a novel purine-based covalent EGFR ligand and a VHL ligand separated by an alkyl chain of seven carbons [240]. CP17 degrades EGFR^L858R/T790M^ and EGFR^del19^ with DC_50_ values of ~ 1 nM and a D_Max_ of ~80%. However, because C797 is the covalent binding site for CP17, this compound is ineffective against C797 mutations. Within the development of CP17, attempts were made to design a reversible covalent EGFR PROTAC by incorporating a cyano group at the α-carbon of the acrylamide and cyclic moieties at the β-carbon, but these modifications perturbed binding affinity. Previously, this group developed the structurally similar noncovalent EGFR degrader P3 (**72**) through cyclopentyl substitution of N9 of the purine scaffold [241]. Intriguingly, epimerization of the VHL ligand in CP17 does not disturb antiproliferative activity in H1975 nor HCC827 cells, although EGFR^L858R/T790M^ and EGFR^del19^ degradation is rescued. E3 ligase ligand competition, EGFR ligand competition, NEDD8 inhibition, and autophagy inhibition were each able to rescue mutant EGFR degradation, but not proteasomal inhibition. These findings suggest that a ubiquitin-lysosome mode of degradation is at play rather than a proteasomal mechanism. Similar observations were made by Qu et al. in 2021 regarding the contribution of lysosomal EGFR degradation in the development of quinazoline EGFR degrader SIAIS125 (**73**) which bears a structurally distinct covalent warhead directed at engaging C797 (compare **73** to **71**) [242].

### Interleukin 1 receptor-associated kinase 4 (IRAK4) degraders

IRAK4 is a S/T kinase located downstream of members of the TLR superfamily [243]. In myelodysplastic syndrome (MDS) and AML, aberrations of mRNA splicing are frequently observed to produce an alternative splice product of IRAK4 which includes exon 4 (IRAK4-L) [244–246]. In contrast to prototypical IRAK4 lacking exon 4 (IRAK4-S), IRAK4-L contains an N-terminal death domain which enables incorporation within the myddosome (Myd88-IRAK4-IRAK2) [247, 248]. While the kinase and scaffolding roles of IRAK4 isoforms within myddosomes remains controversial, IRAK4-L-containing myddosomes are thought to induce oncogenic MAPK and NF-κB signaling via an allosteric interaction with IRAK1 which subsequently interacts with E3 ligase TRAF6 [245, 249]. In hematological malignancies, activating mutations of Myd88 and loss of TLR-axis suppressing miRNAs are thought to play a role in oncogenic myddosome signaling [250, 251]. IRAK4-L expression level negatively correlates with AML prognosis and IRAK4-L abrogation diminishes leukemic cell proliferation [244]. There remain no FDA approved IRAK4 inhibitors, although occupancy-based inhibitors have been reported including Pfizer’s isoquinoline clinical candidate PF-06650833 [252–255].

**Fig. 15:**
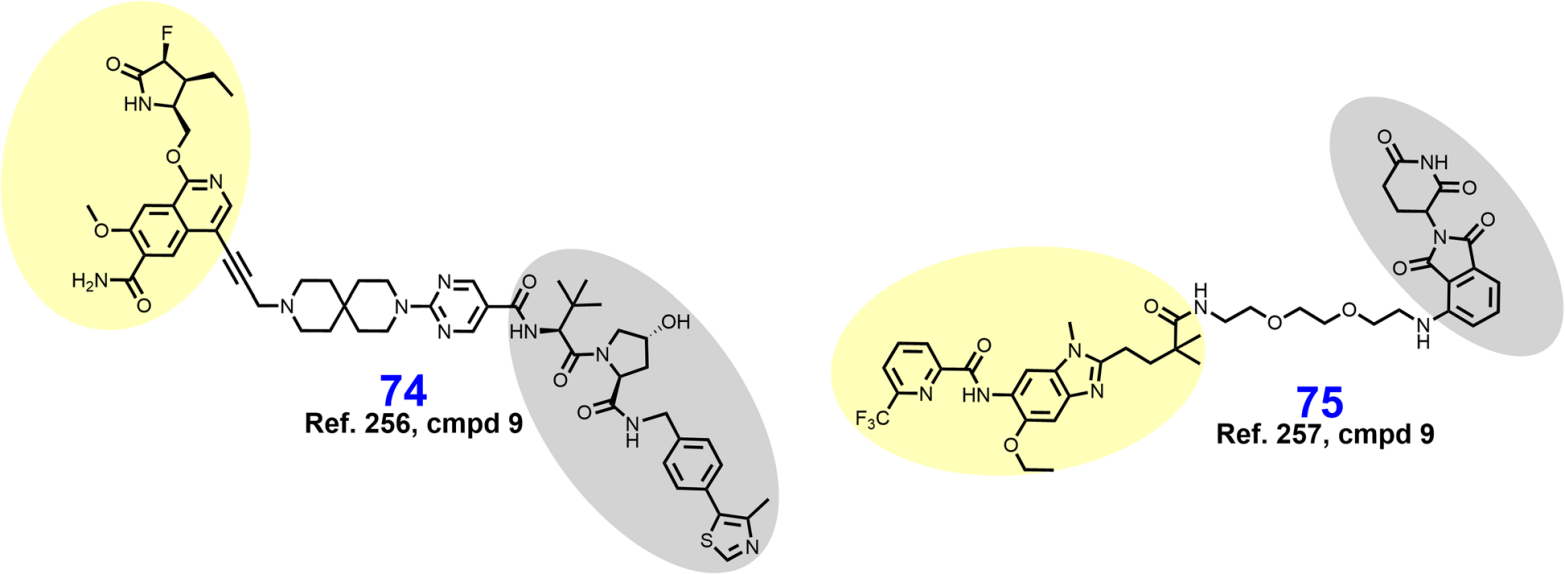
Structures of IRAK4 degraders. Targeting ligands and E3 ligase ligands are highlighted in yellow and gray, respectively

In 2019, Nunes et al. reported the development of VHL-dependent IRAK4 degrader **74** (Fig. 15) after appending PF-06650833 to a rigid, spirocyclic linker incorporating three azaheterocycles [256]. Initially, twelve-atom linear PEG- or alkyl-based linkers were employed to append VHL, CRBN, or cIAP1 ligands to C4 of the isoquinoline core by an ethynyl exit vector. Of this exploratory library, only the alkyl-VHL ligand analogue displayed IRAK4 degradation. Attempts to shorten the twelve-atom alkyl linker abated IRAK4 degradation. The linear alkyl chain was then replaced with the polar, spirocyclic linker depicted in **74**. **74** degrades nearly 100% of IRAK4 in dermal fibroblasts at 1 μM with a DC_50_ of 36 nM. Disappointingly, however, the degradation phenotype did not parallel the IRAK4-null phenotype, as determined by IL-1β-induced IL-6 expression experiments [249, 256].

In 2021, Chen et al. reported their development of CRBN-dependent IRAK4 degrader **75** from a benzimidazole warhead [257]. Initially, a series of alkyl linkers between two and seven carbons in length were utilized to append pomalidomide to the IRAK4 ligand. However, none of the alkyl-iMiD derivatives degraded IRAK4 at 1 μM in MYD88^L265P^ bearing OCI-LY10 and TMD8 cells. A five-atom PEG linker also afforded no IRAK4 degradation, but the eight-atom PEG linker in **75** enabled ~50% degradation of IRAK4 at 1 μM. Mechanistically, **75** produces a dramatic reduction in phosphorylated downstream IκB and NF-κB.

## Degraders of transcriptional regulators

### Bromodomain and extra-terminal domain (BET) degraders

BETs represent one important subfamily of bromodomain-containing epigenetic and transcriptional regulators. Within the BET subfamily are BRDt, BRD1, BRD2, BRD3, and BRD4, the latter of which has been the most well-studied [258, 259]. Via their BD1 and BD2 domains, BETs are recruited to acetylated lysine residues of DNA-bound histones near gene promoters and super-enhancers. There, BETs subsequently recruit p-TEFB and RNA pol II to initiate transcription [258]. Aside from important scaffolding roles in transcription, BRD4 also possesses kinase and histone acetyltransferase (HAT) activities [260, 261]. The HAT activity of BRD4 enables bookmarking of gene expression programs for prompt continuation following mitosis, despite genome-wide dissociation of transcription factors [262]. BRD4 has received considerable attention as a cancer therapy target because several cancers depend on BRD4-mediated c-myc, fos, and aurora kinase B expression [263–265]. A recent report from Devaiah et al, however, warrants close attention before embarking on a BRD4 degrader campaign [266]. Therein, Devaiah et al. describe that occupancy-based displacement of BRD4 from DNA reduces c-myc expression but that BRD4 degradation by a PROTAC paradoxically enhances c-myc levels. The authors, moreover, determine that this a result of loss of BRD4 kinase activity which promotes c-myc ubiquitylation and degradation. JQ1, OTX015, and BI-2536 are occupancy-based inhibitors of BD1 and BD2 DNA-binding that have been reported, although they are known to induce rapid upregulation of BRD4 [265, 267–270]. Myelosuppression, thrombocytopenia in particular, is a known liability of BRD4 inhibition [271, 272]. While most reversible BET inhibitors are nonselective across BD1 and BD2 domains, it has been suggested that domain-specific inhibition of BD1 is a more promising approach to abrogating oncogenic transcription [273].

**Fig. 16:**
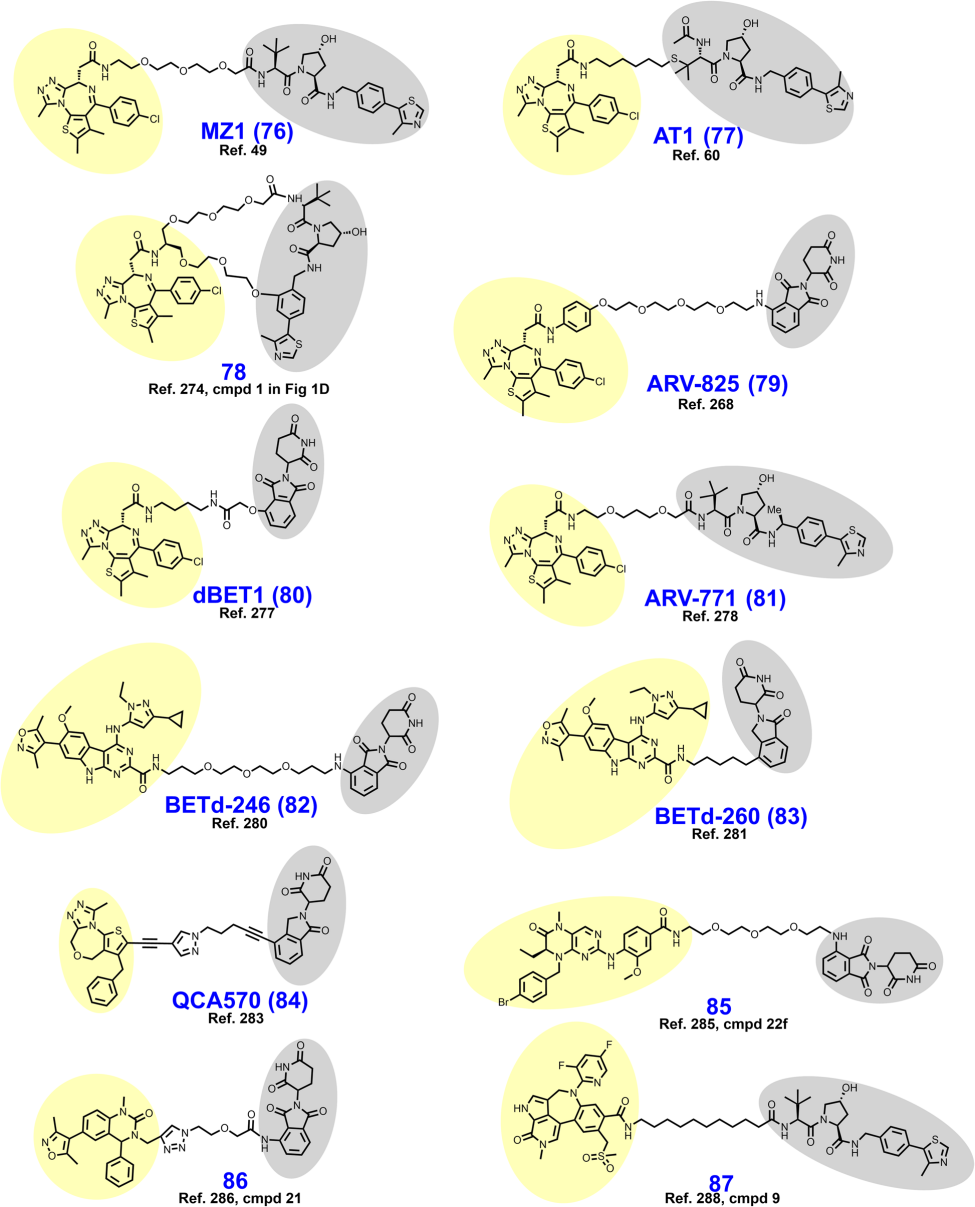
Structures of BET degraders. Targeting ligands and E3 ligase ligands are highlighted in yellow and gray, respectively

**Fig. 17:**
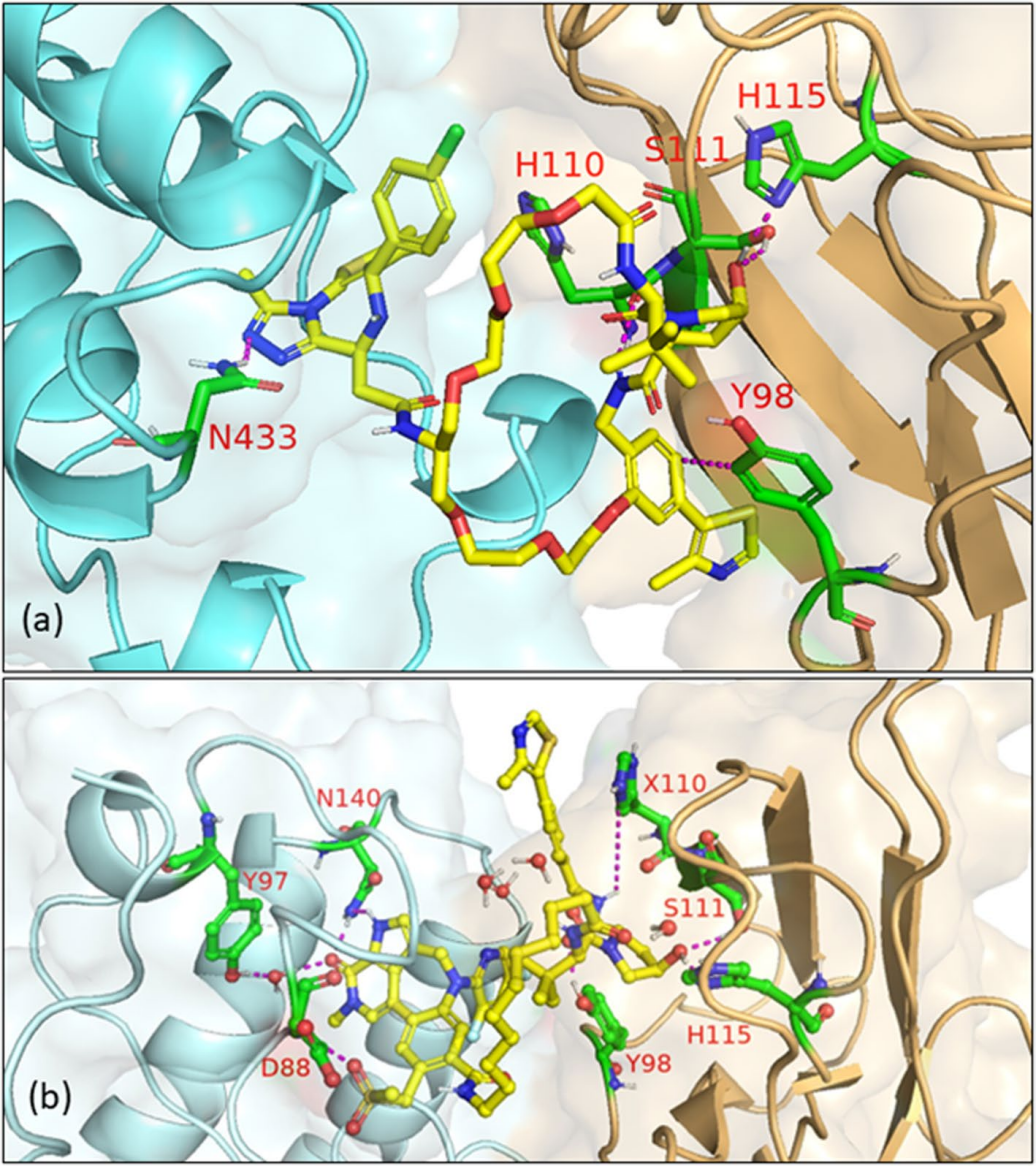
**(a)** Crystal structure of BRD4^BD2^-compound **78**-VHL ternary complex (PDB 6SIS). The binding pattern of 78 nearly superimposes that of MZ1 in complex with Brd4 and VHL (PDB ID: 5T35). Important interactions of this macrocyclic PROTAC include hydrogen bonding with VHL residues Y98, H110, S111, and H115 and BRD4 residues N433 and H437. A T-stacking π - π interaction with VHL^Y98^ is also observed. (**b)** Crystal structure of BRD4^BD1^-Compound **87**-VHL ternary complex (PDB 7KHH). The hydrophobic linker assumes a collapsed conformation to enable intramolecular hydrophobic interactions between the linker, the aryl rings, and the tertbutyl group. Interactions between the ligands and their respective proteins are depicted. Images created using Schrödinger Bioluminate with PDBs 6SIS and 7KHH. BRD4^BD^ subunits are depicted in cyan and VHL in brownish orange

In a landmark 2015 report, Zengerle et al. described the first BET degrader MZ1 (**76**) (Fig. 16), a PROTAC which is furthermore noteworthy as the first PROTAC to employ a non-peptidic VHL ligand [49]. Within this series, the triazolodiazepine BET inhibitor JQ1 was conjugated to a VHL ligand by either three or four PEG units. Zengerle et al. determined that three PEG unit linkers is the preferred linker length by monitoring BET degradation in HeLa and U2OS cells through immunoblotting or image analysis with artificially expressed GFP-BRD4. Moreover, the phenylalanine present in some VHL ligands was shown to reduce BRD4 degradation. Off-target degradation of BRD2 and BRD3 was observed for all reported analogues of MZ1, although other targets of JQ1 were not degraded. Confirmation was furthermore provided that hijacking VHL E3 ligase with chimeric degraders can be achieved without stabilizing the natural substrate HIF-1α. Separately in 2017, Gadd et al. employed MZ1 to solve the first cocrystal structure of a PROTAC in a ternary complex (PDB 5T35) which guided the development of BRD4-selective degrader AT1 (**77**) [60]. Briefly, the crystal structure of MZ1 complexed with BRD4^BD2^-VHL revealed numerous cooperative PPIs and an opportunity to shorten the linker through discarding the tertbutyl group often found in VHL ligands and installing a vector-enabling penicillamine [60]. Later in 2020, Testa et al. cyclized MZ1 with a second PEG chain to obtain the VHL-dependent macrocyclic BRD4 degrader **78** [274]. Compared to MZ1, macrocyclic **78** displays improved selectivity for BRD4^BD2^ relative to BRD4^BD1^ and similar degrader potency despite a 12-fold loss in affinity for BRD4. Testa et al. furthermore succeeded in solving the crystal structure of the ternary complex of BRD4^BD2^-compound **78**-VHL (PDB 6SIS, illustrated in Fig. 17) which provided a structural basis for BRD4^BD2^ selectivity.

In 2015, Lu et al. reported CRBN-dependent BET degrader ARV-825 (**79**) which appends the triazolodiazepine BET inhibitor OXT015 to pomalidomide by a three PEG unit linker [268]. In Namalwa and CA-46 cells, ARV-825 degrades nearly 100% of BRD4 at 3 nM with half-maximal degradation occurring below 1 nM. Overnight ARV-825 exposure produces an accompanying downregulation of c-myc, whereas occupancy-based inhibitors JQ1 and OXT015 triggered rapid BRD4 upregulation and only modest c-myc suppression. Across several B cell lymphoma cell lines, ARV-825 induces substantial increases in caspase 3/7 and cleaved PARP within 24–48 hours. Subsequently, ARV-825’s antiproliferative activity and mechanism of action in AML and ALL models was more extensively probed by Wu et al. and Saenz et al. [275, 276].

In 2015, Winter et al. reported the development of dBET1 (**80**), an in vivo BET degrader wherein a JQ1 analogue is appended to a CRBN ligand by a nine-atom alkylamide linker [277]. Using traditional Western blotting and a novel *in cellulo* immunoblotting technique to assess BRD4 abundance, dBET1 was shown to achieve D_Max_ > 85% with a DC_50_ of 430 nM. A proteomics screen of dBET1-treated MV4;11 cells revealed that BRD2 and BRD3 were depleted to a similar extent as BRD4. In mice inoculated SC or IV with MV4;11 cells, dBET1 treatment induced nearly complete TGI and ~2/3 reduction of bone marrow invasion, respectively.

In 2016, Raina et al. reported BET degrader ARV-771 (**81**) which differs from ARV-825 by employing a VHL ligand, a shorter linker, and discarding the phenyl ring (compare **81** to **79**) [278]. Therein, the rationale for BET degradation in prostate cancer was elegantly demonstrated. AR-driven oncogenic transcription is promoted by a physical interaction between the AR and BRD4, and BRD4 degradation *in cellulo* by ARV-771 depleted both BRD4- and AR-associated transcription products [278, 279]. More intriguingly, BRD4 degradation also depletes the AR and the clinically important AR splice-variant AR-V7 at both the protein and mRNA levels [278]. In vivo, SC administered (30 mg/kg) ARV-771 produced tumor regression or tumor growth inhibition in mice xenografted with 22Rv1 cells and VCaP, respectively.

In 2017, Bai et al. reported their development of CRBN-dependent BET degrader BETd-246 (**82**) and a thorough biological evaluation in triple-negative breast cancer (TNBC) models [280]. Structurally, BETd-246 is composed of the pyrimidoindole targeting ligand BETi-211 appended by a sixteen-atom PEG-dominant linker. In MDA-MB-468 cells, 100 nM BETd-246 rapidly degrades nearly 100% of BRD4 within 1 h. Consistent with immunoblotting experiments, proteomic analysis reveals that BETd-246 degrades BRD2 and BRD3 even more substantially than BRD4. Unlike the parent inhibitor which produces cell cycle arrest predominantly, BETd-246 induced salient apoptosis in a panel of TNBC cell lines. This differential response may be attributable to the pronounced reduction in MCL-1 expression observed with BETd-246 treatment. In vivo, IV administration of BETd-246 displays tumor growth inhibition in mice xenografted with patient-derived and MDA-MB-453 cells, albeit not in other models where tumor penetration was poor.

In 2017, Zhou et al. reported CRBN-dependent BET degrader BETd-260 (**83**) which employs the same pyrimidoindole targeting ligand as BETd-246 [281, 282]. Beginning with an eight-atom alkylamide linker featuring oxyether attachment of the phthalimide, the linker was subsequently shortened to a five-carbon alkyl chain. Exchanging thalidomide for the descarbonyl lenalidomide as the E3 ligase ligand further improved potency and afforded BETd-260. The structural optimization of this BET degrader series was largely guided by WST-8/LDH viability assays in RS4;11 and MOLM-13 cells with only intermittent assessments of BET protein degradation. BETd-260 degrades nearly 100% of BRD2, BRD3, and BRD4 at 1 nM in RS4;11 cells treated for 24 hours. In WST-8 viability assays, BETd-260 achieves IC_50_ values of 51 pM and 2.2 nM in RS4;11 and MOLM-13 cells, respectively. Nanomolar concentrations of BETd-260 downregulate c-myc expression, induce cell cycle arrest, and apoptosis, as determined by Western blotting and flow cytometry, respectively. In vivo, BETd-260 given thrice at 5 mg/kg IV produces complete tumor regression in mice xenografted with RS4;11 cells.

In 2018, Qin et al. described their development of CRBN-dependent BET degrader QCA570 (**84**) based on an oxazepine targeting ligand that confers binding selectivity for BD1 domains relative to BD2 [283]. Therein, alkyl-based linkers ranging from direct attachment to six atoms in length were utilized to append thalidomide or the descarbonyl lenalidomide. Here, a linker length of five-atoms appended to lenalidomide afforded maximal BET degradation. Oxyether, alkyne, secondary amine, and linear alkyl groups were explored as moieties for attachment of the isoindoline ring at the C4 position. By this assessment, it was determined that ethynyl-attachment offered maximal BET degradation. Incorporating these linker SDRs collectively, QCA570 was obtained. In RS4;11 and MV4;11 xenografted mice, QCA570 given intermittently by IV at 1–5 mg/kg produced complete tumor regression.

In 2018, Wang et al. reported CRBN-dependent BET degrader **85** which employs a derivative of the dihydropteridine BI-2536 as a targeting ligand [284, 285]. Guided by WST-8 viability assay data in RS4;11 cells, pomalidomide-appended linkers comprised of alkyl- and PEG-based chains ranging from two to eleven atoms in length were assessed. Bearing the eleven-atom PEG-based linker, **85** degrades nearly 100% of BRD4 in RS4;11 cells at 1 μM and achieves an antiproliferative IC_50_ value of 9.4 nM.

In 2021, Zhang et al. described the development of CRBN-dependent BET degrader **86** based on an analogue of reported dihydroquinazolinone BRD4 inhibitors [286, 287]. Here, PEG- or alkyl-based linkers ranging in length from six to nineteen atoms were employed to append either thalidomide or lenalidomide to N3 of the dihydroquinazolinone scaffold. Within the PEG-based series, a triazole ring was incorporated to enable alkyne-azide click-chemistry. In all derivatives, the isoindoline ring of the CRBN ligand was ligated by amide coupling from the C4 position. After screening their degrader library with an ALPHA-based BRD4 binding assay and a viability assay in HL-60, Raji, and THP-1 cells, the best performing analogue **86** was investigated further. In THP-1 cells treated with 1 μM **86**, approximately 100% of BRD4 is degraded and c-myc is rendered nearly undetectable.

In 2021, Dragovich et al. reported the VHL-dependent BRD4 degrader **87** which employs a pyrrolopyridone targeting ligand that is structurally similar to JQ1 but with improved binding affinity [288, 289]. BRD4 degrader **87** was subsequently tethered to prostate cancer antigen (STEAP1) or AML antigen (CLL1)-targeting antibodies via a GSH-cleavable linker conjugated to the chiral hydroxyl group of the VHL ligand. Dragovich et al. furthermore solved the crystal structure of the VHL-compound **87**-BRD4^BD1^ ternary complex (PDB 7KHH illustrated in Fig. 17). Examining the crystal structure of **87** revealed the ten-carbon alkyl linker to assume a hydrophobic collapse conformation in the ternary complex.

### Signal transducer and activator of transcription 3 (STAT3) degraders

STAT3 is a transcription factor that activates the expression of gene products that govern proliferation, evasion of apoptosis, metastasis, angiogenesis, and an immunosuppressive tumor microenvironment [290–293]. Phosphorylation of STAT3^Y705^ by upstream RTKs (i.e., EGFR, PDGFR, FLT3, BCR-ABL, JAK) has been described canonically to initiate STAT3 activation and STAT3-mediated transcription [291, 294]. In this description, phosphorylation of STAT3^Y705^ enables STAT3 dimerization through interprotein interactions between the transactivation domain (TAD) and the SH2 domain [295, 296]. STAT3 dimers are then imported to the nucleus where they promote chromatin remodeling and bind to gene promoters/enhancers to initiate transcription [294, 297]. The majority of the reported STAT3 inhibitors bind to the SH2 domain to prevent STAT3 dimerization, but some reports suggest that monomers may also independently promote oncogenic transcription [294, 298]. Accordingly, there are concerns about the effectiveness of occupancy-based inhibition of STAT3 dimerization.

**Fig. 18:**
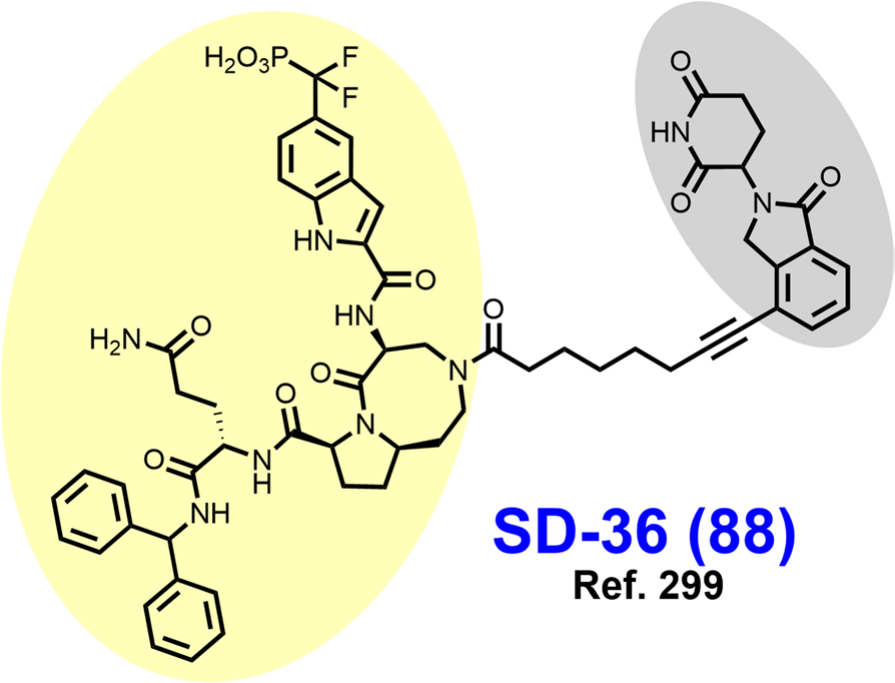
Structure of a STAT3 degrader. Targeting ligands and E3 ligase ligands are highlighted in yellow and gray, respectively

In 2019, Bai et al. reported CRBN-dependent STAT3 degrader SD-36 (**88**) (Fig. 18) based on linker-iMiD derivatization of an optimized analogue of CJ-887 [299, 300]. Structurally, SD-36 employs an eight-atom carbon-based linker which appends the targeting ligand and thalidomide by carboxamide and ethynyl moieties, respectively. In representative AML and ALCL cell lines, 250 nM SD-36 degrades 50–90% of STAT3 and with no observed hook effect below 10 μM. SD-36 displays >20x higher affinity for STAT3 relative to other STAT members and causes no detectable degradation of these isoforms. Nor does SD-36 cause any significant disturbance to other proteins, as determined by proteomics. SD-36 retains the capacity to degrade D661Y, K658R, and Y705F mutation variants of STAT3. In a cell line engineered to express luciferase by a STAT3-controlled promoter, SD-36 reduces luciferase expression with an IC_50_ of 10 nM. In vivo, intermittent IV SD-36 given at 50–100 mg/kg induces complete tumor regression in mice xenografted with MOLM-16, SU-DHL-1, or SUP-M2 cells. Separately in 2021, Hanafi et al. reported their attempt to develop a STAT3 degrader by employing napabucasin as a targeting ligand [301]. However, to their surprise linker-iMiD derivatization instead afforded a degrader of the E3 ligase ZFP91. On this basis, Hanafi et al. determined that while napabucasin downregulates STAT3 and reduces STAT3 phosphorylation, NQO1 and ZFP91 are more likely the true pharmacological targets of this natural product.

## Kirsten rat sarcoma viral oncogene (KRAS) degraders

KRAS is a member of the highly conserved Ras protein superfamily, which includes other clinically relevant paralogs such as HRAS and NRAS [302]. Ras proteins are small GTPases that transduce extracellular growth signals from membrane RTKs to promote cellular proliferation and survival via the MAPK and PI3K signaling pathways among others [303, 304]. KRAS-GTP mediates activation of downstream Raf and PI3K via protein-protein interactions rather than enzymatic activity and is broadly a challenging target to develop ligands for [305–307]. Hydrolysis of bound GTP is ordinarily facilitated by GTPase-activating proteins (GAPs), but cancer-associated G12, G13, and Q61 point mutations render KRAS-GTP invulnerable to GAPs [308–310]

**Fig. 19:**
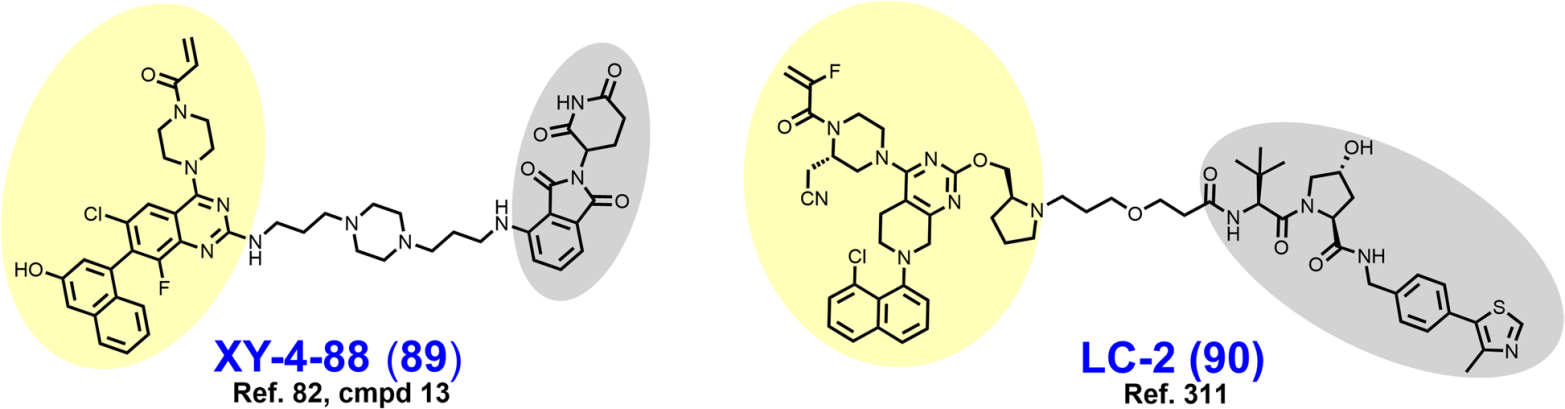
Structures of GFP-KRAS and KRAS Degraders. Targeting ligands and E3 ligase ligands are highlighted in yellow and gray, respectively

In 2020, Zeng et al. reported their development of a CRBN-dependent GFP-KRAS^G12C^ degrader XY-4-88 (**89**) (Fig. 19) after derivatizing a previously reported quinazoline-based covalent G12C inhibitor [82]. However, XY-4-88 neither ubiquitylates nor degrades endogenous KRAS^G12C^. Zeng et al. attribute these disappointing findings to observed differential cellular localization of GFP-KRAS^G12C^ (cytosol) vs. KRAS^G12C^ (membrane/mitochondria)

In 2020, Bond et al. reported VHL-dependent LC-2 (**90**) which employs the FDA approved KRAS^G12C^ inhibitor adagrasib as a targeting ligand [311, 312]. An analogue of LC-2 with a twelve-atom PEG-amide linker was initially evaluated by Bond et al., although this compound afforded no KRAS^G12C^ degradation. Subsequently, it was determined that amides in the linker pose hydrolytic liabilities and that a linker length of ~ 6 atoms was optimal. LC-2 features a six-atom alkoxy linker that appends to the pyrrolidine nitrogen of MRTX849. In NCI-H2030 cells, LC-2 exhibits a D_Max_ of ~ 80% with a DC_50_ of 590 nM. Given that the warhead recruits KRAS^G12C^ by covalent bonding, LC-2 forgoes the benefit of iterative target degradation associated with the PROTAC platform.

## Poly (ADP) ribose polymerase-1 (PARP1) degraders

PARP1 is an enzyme involved in single-stranded DNA break repair (SSB) that when inhibited produces double-stranded DNA breaks (DSBs) after replication of the lesioned DNA [313]. Cancer cells with biallelic BRCA1/2 aberrations exhibit pronounced sensitivity to PARP inhibition relative to BRCA-competent cells [314]. DSBs are unable to be repaired by homology-directed repair (HDR) in BRCA-incompetent cancer cells (−/−) while BRCA-competent noncancerous cells (BRCA +/+ or +/−) retain capacity for HDR. The lethal interaction of PARP inhibition and BRCA-deficiency is currently exploited by FDA approved PARP inhibitors, but resistance (innate or acquired) is commonplace [315]. Nearly half of patients with germline BRCA1/2 mutations fail to respond to PARP inhibitors (PARPis) [316, 317].

**Fig. 20:**
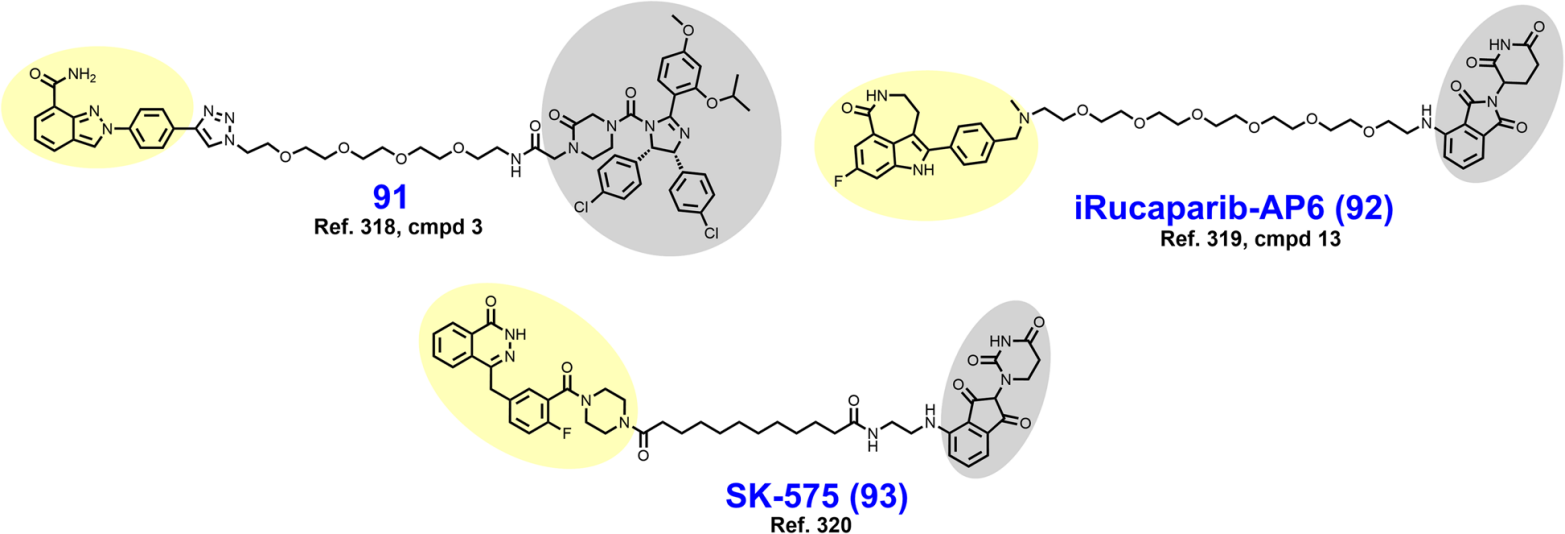
Structures of PARP1 degraders. Targeting ligands and E3 ligase ligands are highlighted in yellow and gray, respectively

In 2019, Zhao et al. reported the MDM2-dependent PARP1 targeted chimera **91** (Fig. 20) through utilizing a derivative of the indazole PARP inhibitor niraparib as a targeting ligand [318]. **91** features a seventeen atom PEG-amide based linker with a triazole ring replacing the piperazine ring seen in niraparib. **91** induces PARP1 cleavage which is reversible by niraparib competition, proteasomal inhibition, and NAE inhibition, but it remains unclear whether PARP1 is a degradative target or if degradation of an alternative target instead induces apoptosis.

In 2019, Wang et al. synthesized CRBN-dependent PARP1 degrader iRucaparib-AP6 (**92**) for the purpose of developing therapeutics that inhibit PARP1 enzymatic activity without PARP trapping [319]. Prior to this report, the relative contributions of PARylation inhibition vs. PARP trapping at SSBs toward the anticancer effects of PARPis were unknown. Inhibition of PARylation without inducing lethal PARP trapping holds high clinical value in ischemia-reperfusion injury and neurodegenerative disorders because PAR chains trigger a form of cell death known as parthanatos. However, clinically approved PARPis cannot achieve this without simultaneous PARP trapping. iRucaparib-AP6 abates PARylation without causing PARP trapping, as determined by fluorescent microscopy and subfraction immunoblotting.

In 2020, Cao et al. reported the development of CRBN-dependent PARP1 degrader SK-575 (**93**) after derivatizing an analogue of the phthalazinone PARP1 inhibitor olaparib [320]. SK-575 discards the cyclopropyl group of olaparib to append a phthalimide by a fifteen atom alkylamide linker. Exploration of linker SDRs for this set of ligands focused on varying alkylamide linker length between six and seventeen atoms, although PEG-based linkers, VHL ligands, and ether-attached CRBN ligands were evaluated. In MDA-MB-436 and SW620 cells, SK-575 degrades PARP1 with a D_Max_ of > 99% and DC_50_ values of ~ 1 nM. Mechanistically, in BRCA1/2 (−/−) cells SK-575 induces formation of γH2AX foci more potently than olaparib. In vivo, SK-575 given IP achieves tumor growth inhibition in mice xenografted with Capan-1 cells (BRCA2 −/−), whereas combination therapy with temozolomide was required in the BRCA-competent SW620 xenograft model.

## Src homology 2 domain-containing protein tyrosine phosphatase (SHP2) degraders

SHP2 is a tyrosine-specific phosphatase (PTP) encoded by the PTPN11 gene which functions as a critical node between membrane RTKs and downstream signaling pathways [321, 322]. In the resting state, the catalytic PTP domain of SHP2 is autoinhibited by the N-SH2 and C-SH2 domains [322]. SH2 domain binding of phosphorylated sites in the intracellular domain of RTKs activates SHP2 PTP activity through relief of autoinhibition [322]. The catalytic site of SHP2 is particularly difficult to drug directly owing to the pocket’s shallow and positively charged character [84]. While docked at the intracellular domain of RTKs, SHP2 interacts with Sos1-binding partners to promote KRAS nucleotide cycling and subsequent MAPK signaling [321, 323]. SHP2 also promotes an immunosuppressive tumor microenvironment by transducing signaling from PD-1 receptors on lymphocytes [324]. While SHP2 mutations are found in certain hematological malignancies, the rationale for drugging SHP2 is often because of its location at the interface of transmembrane RTKs and intracellular signal transduction pathways (i.e., MAPK, JAK/STAT, PI3K-AKT) [325, 326].

**Fig. 21:**
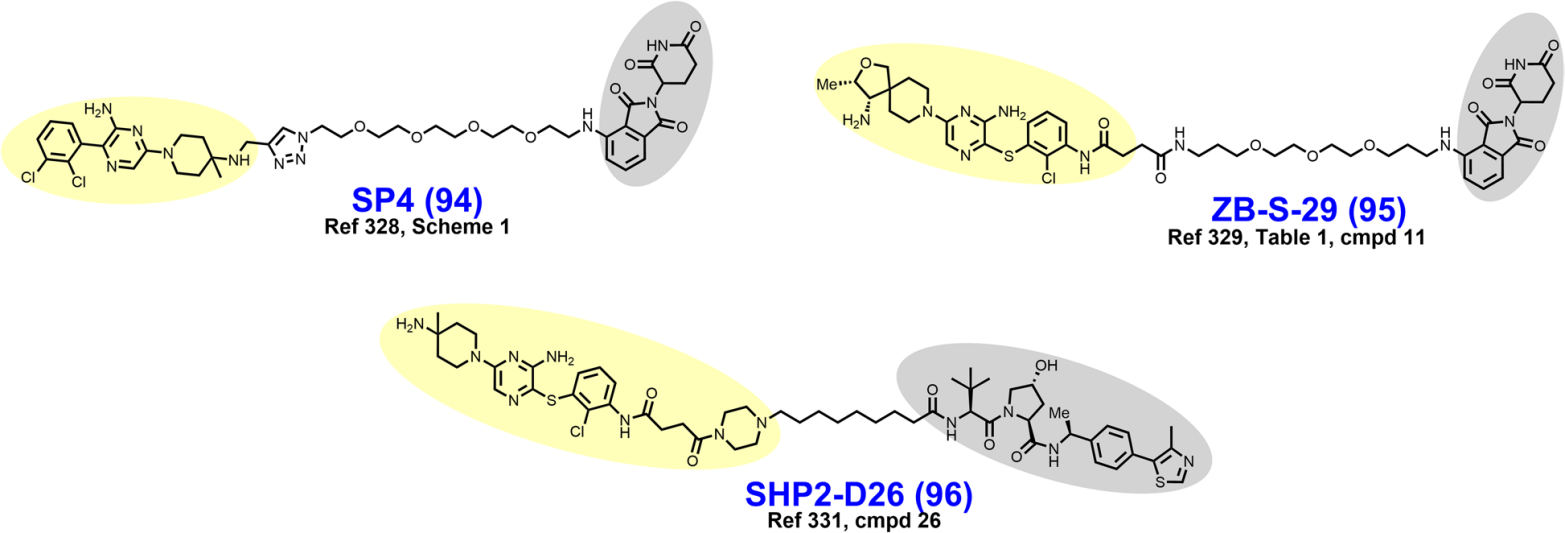
Structures of SHP2 degraders. Targeting ligands and E3 ligase ligands are highlighted in yellow and gray, respectively

In 2021, Zheng et al. reported their development of CRBN-dependent SHP2 degrader SP4 (**94**) (Fig. 21) through linker-iMiD derivatization of the pyrazine allosteric inhibitor SHP099 [327, 328]. Structurally, SP4 appends pomalidomide by a four PEG-containing linker with a triazole ring proximal to SHP099 to enable click chemistry. Derivatization within their series included varying the number of PEG units contained within the linker. In HeLa cells, SP4 degrades SHP2 in the double-digit nanomolar range, although depletion was modest unless 48–96 hours of incubation were implemented. Compared to its parent inhibitor, SP4 more robustly induces apoptosis and with 10-fold lower potency, as determined by flow cytometry.

In 2021, Yang et al. reported the development of CRBN-dependent SHP2 degrader ZB-S-29 (**95**) after exploring linker-iMiD derivatization of a derivative of the pyrazine allosteric SHP2 inhibitor TNO155 [329, 330]. Therein, PEG- and alkyl-based linkers affixed to either C4 or C5 of the iMiD were assessed by immunoblotting in MV4;11 cells. Broadly, PEG linkers induced higher SHP2 degradation than alkyl linkers, longer linker length were preferred, and the attachment point on the isoindoline ring was inconsequential. ZB-S-29 features a PEG-amide linker twenty atoms in length where the isoindoline is appended by a secondary amine. In MV4;11 cells, ZB-S-29 displays a D_Max_ exceeding 90% with a DC_50_ of 6.02 nM and more robustly induces apoptosis than SHP099.

In 2020, Wang et al. reported the development of VHL-dependent SHP2-D26 (**96**) based on a derivative of the pyrazine allosteric inhibitor SHP099 [327, 331]. Linkers ranging in length from eight to nineteen atoms were assessed, but those shorter than thirteen atoms afforded minimal SHP2 degradation in KYSE520 cells. A nineteen atom piperazine-containing alkylamide is featured in SHP2-D26. In KYSE-520 and MV4;11 cells, SHP2-D26 achieves D_Max_ exceeding 95% with single-digit nanomolar DC_50_ values. Compared to SHP099, SHP2-D26 is approximately two logarithmic orders more potent at reducing phosphorylation of downstream ERK.

## Miscellaneous degraders of SMARCA2/4, Bcl-xL, and WDR5

**Fig. 22:**
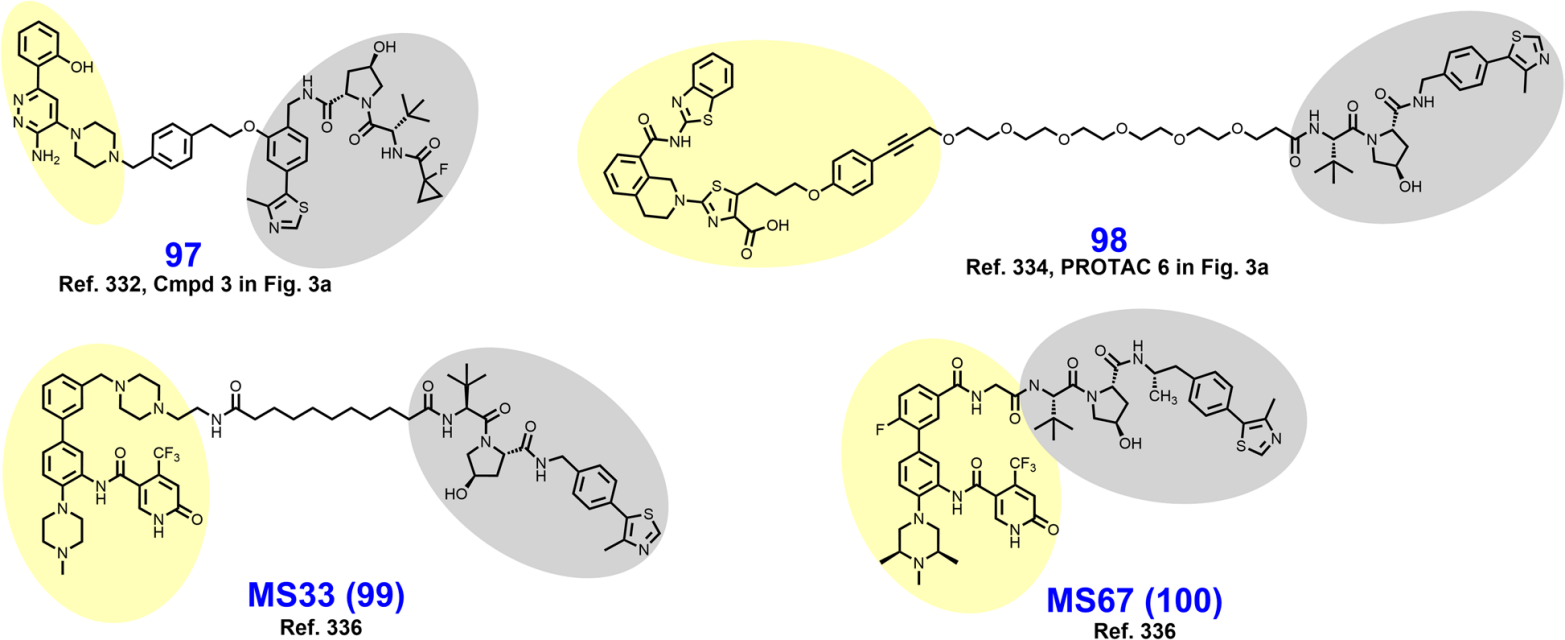
Structures of miscellaneous degraders. Targeting ligands and E3 ligase ligands are highlighted in yellow and gray, respectively

**Fig. 23:**
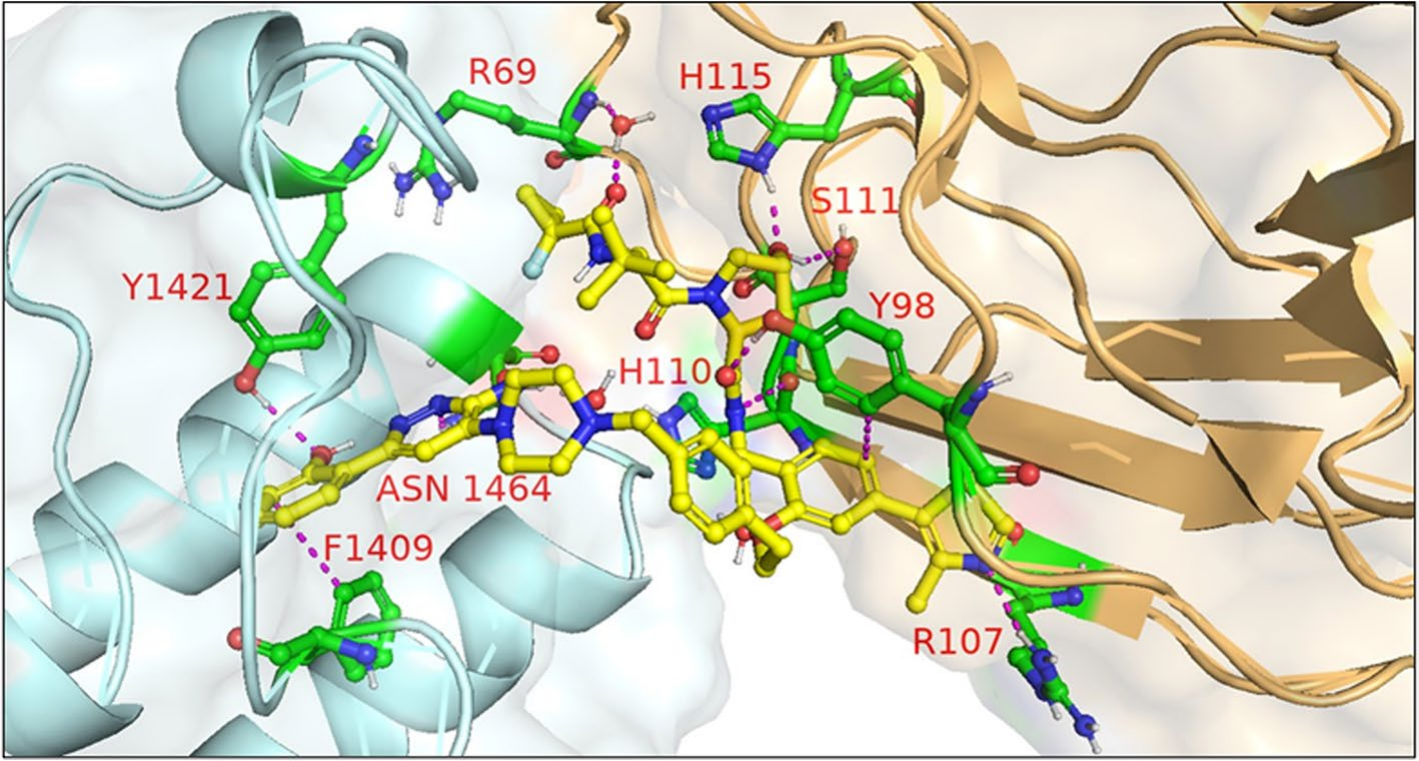
Crystal structure of VHL-compound **97**-SMARCA2^BD^ ternary complex (PDB 6HAX). The crystal structure depicts a sought after T-stacking interaction between the linker aryl group and VHL^Y98^. VHL^R69^ forms critical interactions with the backbone of SMARCA2^BD^ residues and a water-mediated hydrogen bond to the VHL ligand. VHL^R69A^ mutations dramatically reduce degrader potency. Additional ligand-protein interactions are depicted. Image created using Schrödinger Bioluminate with PDB 6HAX. SMARCA2^BD^ is depicted in cyan and VHL in brownish orange

In 2019, Farnaby et al. reported their development of VHL-dependent SMARCA1/2 degrader **97** (Fig. 22) by derivatizing an aminopyridazine SMARCA^BD^ targeting ligand with an alkylaromatic linker [332]. The VHL ligand of **97** is distinguished by a cyclopropylfluoryl group [333] and phenoxy attachment of the linker. PEG-based analogues of **97** promoted cooperative ternary complex formation, although their cellular permeability was limited. On this, basis **97** was designed with the intent of gaining a T-stacking interaction with VHL^Y98^ by the aryl linker group and reducing linker polarity. Farnaby et al. solved the crystal structure of the ternary complex of VHL-compound **97**-SMARCA2^BD^ (PDB 6HAX illustrated in Fig. 23) which depicted the desired π-π interaction and preservation of cooperative interactions seen in their earlier crystal structures (i.e., PDB 6HAY). Proteomics studies revealed that **97** degrades a handful of members of SWI/SNF chromatin-remodeling complexes including SMARCA2/4.

**Fig. 24:**
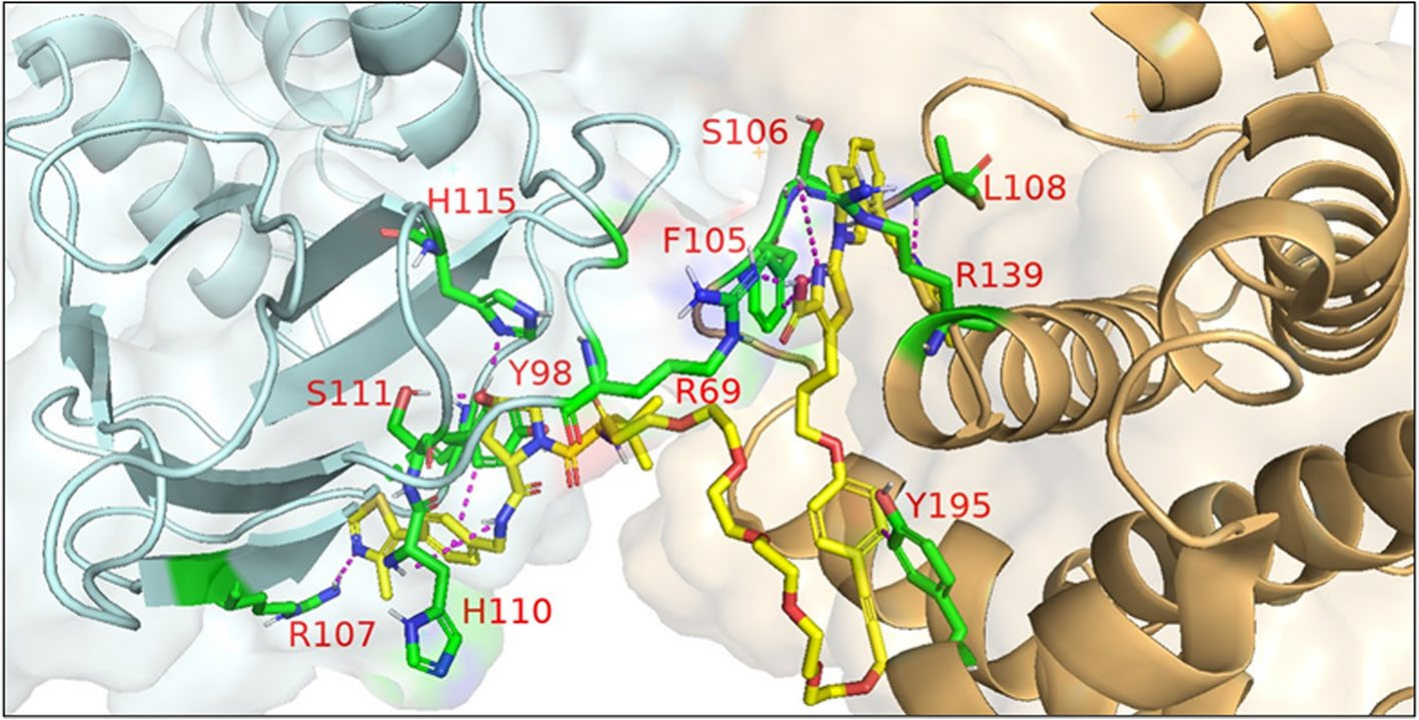
Crystal structure of VCB-compound **98**-Bcl-xL ternary complex (PDB 6ZHC). The PEGylated linker folds back after exiting the Bcl-xL binding pocket via the ethynyl exit vector. Image created using Schrödinger Bioluminate with PDB 6ZHC. VHL is depicted in cyan and Bcl-xL in brownish orange

In 2020, Chung et al. described the development of VHL-dependent Bcl-xL degrader **98** through derivatization of a tetrahydroisoquinoline Bcl-xL inhibitor [334, 335]. Therein, the crystal structure of the VCB-compound **98**-Bcl-xL ternary complex (PDB 6ZHC illustrated in Fig. 24) was solved. In this structure, the PEG linker folds back onto itself immediately after the ethynyl exit vector clears the α3-, α4-helix groove of Bcl-xL. Despite the negative cooperativity of ternary complex formation, **98** degrades ~76% of BcL-xL at a concentration of ~300 nM in THP-1 cells treated for 24h.

**Fig. 25:**
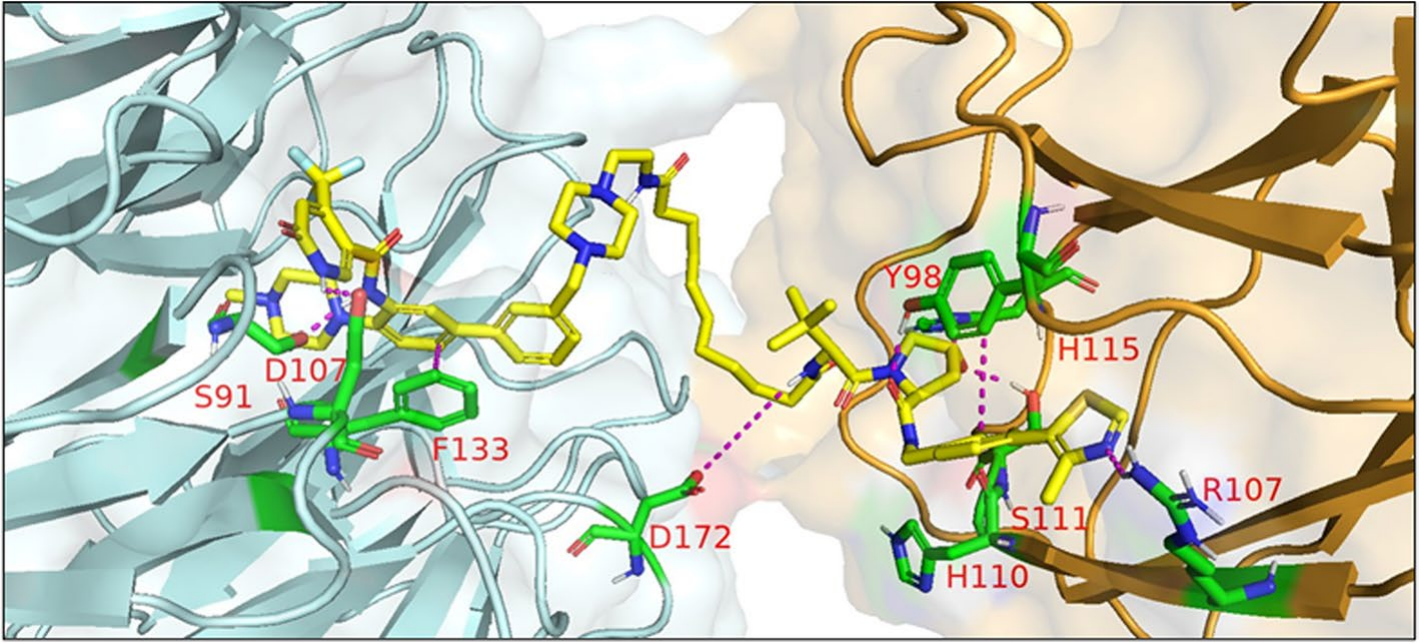
Crystal structure of VCB-MS33-WDR5 ternary complex (PDB 7JTO). Image created using Schrödinger Bioluminate with PDB 7JTO

In 2021, Yu et al. reported the discovery of VHL-dependent WDR5 degrader MS33 (**99**) through derivatizing a previously reported WDR5-MLL interaction modulator [336, 337]. Broadly, PEG-based linkers afforded minimal WDR5 degradation in MV4;11 cells when paired with a VHL ligand whereas alkyl-based congeners depleted WDR5 when length exceeded six atoms. Yu et al. solved the crystal structure (illustrated in Fig. 25) of the VCB-MS33-WDR5 complex, revealing an extended linker conformation and an opportunity to expand the VHL-WDR5 protein-protein interface. On this basis, the in vivo WDR5 degrader MS67 (**100**) was synthesized by discarding the linker-attached piperazine ring, connecting the ethylamine exit vector directly to the VHL ligand, and subtly derivatizing the targeting ligand (compare **100** to **99**) [336].

## Conclusion and future directions

Oncoproteins represent therapeutic targets that remain challenging to approach with occupancy-based drug design. Clinically approved molecularly targeted therapies are often efficacious initially when given to patients harboring the appropriate tumor histology and genetic profile. However, the durability of these agents is limited by factors related to leaving the target intact, requirements for high binding affinity, and the propensity for cancer cells to adaptively spawn resistant variants of the target. PROTACs induce TPD by hijacking E3 ligases to ubiquitylate neosubstrate targets in an event-driven MOA. While the cellular permeability hurdle has been cleared, it remains an important consideration at all stages of PROTAC design given their high MWs. The PROTAC platform offers several distinct advantages over occupancy-based inhibitors including (i) less stringent requirements for binding affinity, (ii) target degradation opposed to stabilizing an inactive conformation, (iii) substoichiometric, iterative target degradation, (iv) opportunities to improve selectivity for targets with conserved binding sites across related isoforms, (v) ability target the dark proteome including transcription factors and scaffolding proteins, and (vi) resiliency to point mutations. Beyond their value as therapeutics, PROTACs have high value as chemical knockdown tools because of their exquisite selectivity, rapid effects, and capacity to elucidate the MOA of the parent ligand (i.e., PARP trapping vs. PARylation inhibition). The medicinal chemistry of PROTAC design remains a challenging area of research because the SDRs for a pair of ligands are typically determined empirically by extensive chemical synthesis. Methods are now available for rapidly synthesizing an exploratory library of PROTACs spanning a diverse chemical space, followed by biological evaluation without chromatography.

There have been many lessons learned in recent years that will pave the way for successful degrader campaigns in the future. First, short linkers are liable to steric clashes between the interacting proteins, and thermodynamic cooperativity of ternary complex formation is not strictly required. Regardless of length, certain linker compositions (i.e., linear alkyl chain or PEG-based) can be strictly incompatible for a given pair of E3 ligase- and target-recruiting ligands. Additionally, both E3 ligase expression in the target tissue and E3 ligase-target compatibility are factors that should be determined early on. Collectively, these findings suggest that degrader campaigns should begin with an assessment of E3 ligase expression in the target tissue followed by querying several E3 ligase ligands appended by long linkers of various compositions. An additional recommendation is to query several distinct targeting ligands early in the medicinal chemistry campaign. Targets that are localized to the membrane or the nucleus can pose challenges for initial assessments of an exploratory degrader library. Here, use of a model cell line engineered to express a recombinant target that is cytosolically-localized may offer a way to examine SDRs before later refining the subcellular pharmacokinetics of the degrader.

A method for evaluating the suitability of a target for the PROTAC platform has now been described and uncovered over a thousand candidate targets that have no reported degraders. However, degrader development for these targets may be difficult without a larger set of validated E3 ligases with corresponding drug-like ligands. Identification of E3 ligases that are suitable for the PROTAC platform and discovery of their ligands remains an active area of research. While the PROTACs reviewed here were designed based on repurposing occupancy-based inhibitors for degrader development, the development of targeting ligands specifically for the purpose of PROTAC design remains underexplored. The optimal targeting ligand for a PROTAC is not necessarily one which displays the highest binding affinity, nor the most potent enzymatic inhibition. Other properties of targeting ligands such as binding in proximity to surface lysine residues or near surfaces predicted to form favorable PPIs with the recruited E3 ligase may also be of importance.

While still challenging to obtain, several cocrystal structures of PROTAC-induced ternary complexes have now been reported. High resolution structures of ternary complexes have enabled reductions in MW through shortening or repositioning of the linker and removing unnecessary atoms in the ligands [60, 64]. Crystal structures may additionally offer a basis for rationalizing target-E3 ligase compatibility/incompatibility, as well as provide actionable insights regarding optimal linker composition and positions where rigidity is tolerated. Advances in computational modeling of protein-protein interactions and ligand-induced ternary complexes will furthermore usher in an era of structure-based PROTAC design.

## Data Availability

Not applicable.
